# Genomic co-alteration patterns are associated with differential benefit from first-line third-generation EGFR-TKI plus chemotherapy versus TKI monotherapy in advanced EGFR-mutant NSCLC

**DOI:** 10.3389/fmed.2026.1969669

**Published:** 2026-10-21

**Authors:** Lili Bai, Huijie Wu, Yue Jiang, Dan Ran, Ying Li, Jianxi Zhou, Teng Duan

**Affiliations:** 1 Department of Western Pharmacy, Cangzhou Hospital of Integrated Traditional Chinese and Western Medicine-Hebei, Cangzhou, Hebei, China; 2 Department of Chemoradiotherapy and Hematology, Chengde Central Hospital, Chengde, Hebei, China; 3 Department of Hematological Oncology, Cangzhou Hospital of Integrated Traditional Chinese and Western Medicine-Hebei, Cangzhou, Hebei, China; 4 Department of Interventional and Breast Medical Oncology, Cangzhou Hospital of Integrated Traditional Chinese and Western Medicine-Hebei, Cangzhou, Hebei, China; 5 Department of Head, Neck and Thoracic Oncology, Cangzhou Hospital of Integrated Traditional Chinese and Western Medicine-Hebei, Cangzhou, Hebei, China; 6 National Clinical Research Center for Cancer, Key Laboratory of Cancer Prevention and Therapy, Tianjin Medical University Cancer Institute and Hospital, Tianjin, China

**Keywords:** chemotherapy, co-mutation, EGFR mutation, non-small-cell lung cancer, propensity score matching, third-generation TKI

## Abstract

**Background:**

Third-generation EGFR tyrosine kinase inhibitors (EGFR-TKIs) plus pemetrexed-platinum chemotherapy are a first-line option for advanced EGFR-mutant NSCLC. However, whether benefit varies by genomic co-alteration background remains unclear. This study evaluated whether baseline co-alteration patterns are associated with differential efficacy of combined chemotherapy versus TKI monotherapy.

**Methods:**

We retrospectively enrolled patients with advanced EGFR-mutant NSCLC receiving first-line third-generation EGFR-TKI monotherapy or combination with pemetrexed-platinum chemotherapy at two centers (2018–2025). Based on baseline NGS, patients were stratified into group A (EGFR mutation alone), group B (non-driver co-mutations), and group C (driver co-mutations). One-to-one propensity score matching (PSM) was applied. The primary endpoint was progression-free survival (PFS); secondary endpoints included overall survival (OS), objective response rate (ORR), intracranial efficacy, and safety.

**Results:**

Overall, 848 patients were enrolled; after PSM, 285 remained in each arm. The combination arm showed numerically longer median PFS (23.3 vs. 17.3 months; HR = 0.654; *P* < 0.001) and OS (41.2 vs. 33.7 months; HR = 0.750; *P* = 0.005). Group B derived the greatest benefit (mPFS: 23.0 vs. 15.3 months, HR = 0.554, *P* < 0.001; mOS: 39.9 vs. 33.1 months, HR = 0.694, *P* = 0.007), whereas groups A (mPFS: 26.5 vs. 22.8 months, HR = 0.833, *P* = 0.253) and C (mPFS: 14.5 vs. 10.2 months, HR = 0.584, *P* = 0.088) showed no significant differences. The treatment-by-co-alteration interaction for PFS was significant (*P* = 0.032). Among patients with baseline brain metastases, median intracranial PFS was numerically longer with combination therapy than with monotherapy (18.8 vs. 12.6 months; HR = 0.739, 95% CI 0.544–1.003; log-rank *P* = 0.047), although the difference was of borderline statistical significance. Hematologic toxicities were more frequent with combination therapy but manageable.

**Conclusions:**

In advanced EGFR-mutant NSCLC, baseline co-alteration patterns are associated with the magnitude of benefit from first-line combined chemotherapy. Patients with non-driver co-mutations may derive greater PFS and OS benefit from TKI plus chemotherapy, whereas those with EGFR mutation alone fare well with TKI monotherapy. Given the retrospective design, biological heterogeneity within subgroups, small driver-co-mutation sample size, and non-significant OS interaction, these findings are hypothesis-generating and require prospective validation before guiding individualized treatment decisions.

## 1 Introduction

Lung cancer is the leading cause of cancer-related mortality worldwide, and non-small-cell lung cancer (NSCLC) accounts for approximately 80%–85% of all lung cancer cases (1). Epidermal growth factor receptor (EGFR) gene mutations represent one of the most important actionable driver alterations in NSCLC, occurring in roughly 40%–50% of Asian patients with lung adenocarcinoma—a proportion substantially higher than that observed in Western populations (2). The discovery of EGFR-sensitizing mutations, predominantly exon 19 deletions and the exon 21 L858R point mutation, has fundamentally transformed the treatment landscape of advanced NSCLC, and EGFR tyrosine kinase inhibitors (TKIs) have become the standard of care in the first-line setting for these patients (3).

Third-generation EGFR-TKIs selectively target EGFR-sensitizing mutations as well as the EGFR T790M resistance mutation while sparing wild-type EGFR, thereby demonstrating superior efficacy and safety profiles relative to first- and second-generation agents (4). The landmark FLAURA trial established osimertinib as first-line standard therapy by showing that, in previously untreated patients with EGFR-mutant advanced NSCLC, osimertinib significantly prolonged both progression-free survival (PFS; median 18.9 vs. 10.2 months; HR, 0.46) and overall survival (OS; median 38.6 vs. 31.8 months; HR, 0.80) compared with first-generation TKIs (gefitinib or erlotinib) (4, 5). Subsequently, the domestic third-generation TKIs aumolertinib and furmonertinib also demonstrated PFS superiority over gefitinib in the AENEAS and FURLONG studies, respectively, further expanding clinical treatment options (6, 7).

Nonetheless, even with third-generation TKI monotherapy, the vast majority of patients experience disease progression within 1–2 years, and acquired resistance remains inevitable (8). To further delay resistance and improve long-term outcomes, several studies have explored strategies combining TKIs with chemotherapy. The FLAURA2 trial showed that adding pemetrexed-platinum chemotherapy to osimertinib extended median PFS from 16.7 to 25.5 months (HR, 0.62; *P* < 0.001) and also significantly improved central nervous system (CNS) efficacy (9, 10). Similarly, the AENEAS2 and ACROSS2 studies confirmed PFS benefit with aumolertinib plus chemotherapy relative to monotherapy (11, 12). These data have propelled the clinical adoption of combination regimens; at the same time, however, chemotherapy is associated with significantly increased hematologic and non-hematologic toxicities, underscoring the importance of identifying the populations most likely to benefit from combined treatment.

Accumulating evidence indicates that EGFR-mutant tumors are not a homogeneous disease entity, and the presence of baseline co-alterations profoundly influences TKI efficacy and resistance patterns (13). TP53 mutations are the most common co-alteration in EGFR-mutant NSCLC, occurring in approximately 50%–60% of cases; multiple studies have linked TP53 co-mutations to shorter PFS and OS and to the earlier emergence of resistant clones (14, 15). In addition, alterations such as PIK3CA amplification or mutation, RB1 loss, and CDKN2A deletion have been associated with inferior TKI outcomes (16–18). Critically, when EGFR mutations coexist with other driver gene alterations (e.g., MET amplification, ALK fusion, or KRAS mutation), the tumor biology is more aggressive and the efficacy of TKI monotherapy is often limited (13). Although prior studies have examined the impact of specific co-alterations on prognosis with TKI monotherapy, there remains a paucity of systematic investigation into how co-alteration patterns influence the relative benefit of TKI plus chemotherapy. Biologically, tumors harboring non-driver co-mutations (such as TP53 or PIK3CA) may exhibit greater genomic instability and clonal heterogeneity, and the cytotoxic effect of chemotherapy could help eliminate TKI-insensitive subclones, thereby delaying resistance. In contrast, for tumors with driver co-mutations, the simple addition of chemotherapy may be insufficient to overcome resistance mediated by bypass pathway activation. On the other hand, patients with EGFR mutation alone can achieve durable responses with TKI monotherapy, and the incremental benefit of adding chemotherapy may be limited.

Against this background, we conducted a two-center retrospective study enrolling 848 patients with advanced EGFR-mutant NSCLC who received first-line third-generation EGFR-TKI monotherapy or combined chemotherapy. We applied propensity score matching (PSM) to balance between-group differences and performed stratified analyses based on baseline NGS co-alteration profiles. The primary objective was to elucidate whether genomic co-alteration patterns are associated with the differential benefit of combined chemotherapy versus monotherapy, thereby generating hypotheses regarding the potential utility of co-alteration profiling in EGFR-mutant NSCLC.

## 2 Materials and methods

### 2.1 Study design and patient population

This was a two-center, retrospective, observational cohort study. Data were sourced from the electronic medical record systems and tumor follow-up registries of Tianjin Medical University Cancer Institute and Hospital and Cangzhou Hospital of Integrated Traditional Chinese and Western Medicine. The study protocol was reviewed and approved by the Ethics Committee of Cangzhou Hospital of Integrated Traditional Chinese and Western Medicine-Hebei (Cangzhou, China; approval no. CZX2024180) and the Institutional Review Board of Tianjin Medical University Cancer Institute and Hospital (Tianjin, China; approval no. EK20250091). Given the retrospective nature of the study, the requirement for informed patient consent was waived. The study was designed and reported in strict accordance with the Declaration of Helsinki and the Strengthening the Reporting of Observational Studies in Epidemiology (STROBE) guidelines (19).

#### 2.1.1 Inclusion and exclusion criteria

Inclusion criteria:

(1) Histologically or cytologically confirmed advanced NSCLC (stage IV); (2) EGFR-sensitizing mutation—i.e., exon 19 deletion (19del) or exon 21 L858R point mutation—verified by amplification refractory mutation system PCR (ARMS-PCR) or next-generation sequencing (NGS); (3) age ≥ 18 years; (4) Eastern Cooperative Oncology Group (ECOG) performance status score of 0–2; (5) receipt of first-line third-generation EGFR-TKI (osimertinib, aumolertinib, or furmonertinib) monotherapy or third-generation EGFR-TKI combined with pemetrexed-platinum chemotherapy between January 2018 and December 2025; (6) at least one measurable lesion at baseline, as defined by Response Evaluation Criteria in Solid Tumors (RECIST) version 1.1 (20); and (7) availability of complete baseline clinical data, imaging assessment records, and follow-up data.

Exclusion criteria:

(1) Prior systemic anticancer therapy for advanced disease (including chemotherapy, targeted therapy, or immunotherapy); (2) concurrent history of other malignancies or a second primary tumor; (3) missing baseline NGS data or inability to perform co-alteration stratification; (4) pregnancy or lactation; (5) follow-up duration of less than 3 months without recorded disease progression or death; and (6) severely incomplete clinical records that would preclude efficacy or safety evaluation.

#### 2.1.2 Baseline data collection

Baseline clinical data were collected through the electronic medical record system, including age, sex, smoking history (never smoker vs. former/current smoker), ECOG performance status score, EGFR mutation subtype (19del vs. L858R), metastatic sites (brain, liver, bone, adrenal gland, pleura, etc.), co-alteration grouping and co-mutation burden, TKI type (osimertinib/aumolertinib/furmonertinib), and NGS sample type (tissue vs. plasma). Data regarding prior or concurrent radiotherapy, including cranial radiotherapy for brain metastases and palliative thoracic radiotherapy, were also recorded when available. For patients in the combination arm, the chemotherapy regimen (carboplatin vs. cisplatin), number of chemotherapy cycles, receipt of pemetrexed maintenance therapy, and synchronous versus sequential initiation of TKI and chemotherapy were additionally recorded. All baseline data were independently extracted and cross-checked by two trained clinical researchers; discrepancies were adjudicated by a senior investigator.

### 2.2 Group definitions and treatment regimens

Treatment allocation was not randomized and was determined at the discretion of the treating physicians in consultation with each patient, based on individualized clinical assessment. Factors that may have influenced treatment choice included disease burden, symptomatic disease, CNS disease severity, comorbidities, organ function and chemotherapy tolerance, physician and patient preference, and the prevailing clinical guidelines at the time of treatment initiation, among other clinical considerations. No formal randomization or protocol-driven allocation was applied. Patients were divided into two groups according to the first-line treatment regimen: the combination group (third-generation EGFR-TKI plus pemetrexed-platinum chemotherapy) and the monotherapy group (third-generation EGFR-TKI alone). In the combination regimen, the TKI was administered orally at the standard dose (osimertinib 80 mg once daily, aumolertinib 110 mg once daily, or furmonertinib 80 mg once daily). Chemotherapy consisted of carboplatin (AUC = 5, every 21 days) or cisplatin (75 mg/m^2^, every 21 days) combined with pemetrexed (500 mg/m^2^, every 21 days) for 4–6 cycles. After completion of chemotherapy, continued TKI with or without pemetrexed maintenance was permitted until disease progression, intolerable toxicity, or patient refusal. The TKI and chemotherapy were initiated within the same treatment cycle (interval ≤ 7 days). Patients in the monotherapy group received standard-dose third-generation EGFR-TKI alone until disease progression or intolerance. Radiotherapy, including cranial radiotherapy for brain metastases and palliative thoracic radiotherapy, was permitted at the discretion of the treating physician and was not part of the defined study intervention.

Based on baseline NGS results, patients were further classified into three co-alteration subgroups: group A (EGFR mutation alone), defined as the absence of other clinically meaningful gene alterations beyond the EGFR-sensitizing mutation; group B (concomitant non-driver co-mutations), defined as the presence of alterations in non-driver genes such as TP53, CDKN2A/B, PIK3CA, RB1, CTNNB1, ARID1A, CCND1/2/3, PTEN, STK11, ATM, SETD2, or SMARCA4, without known oncogenic driver gene alterations; and group C (concomitant driver co-mutations), defined as the presence of driver gene alterations including KRAS mutation, MET amplification, ERBB2 (HER2) amplification or mutation, BRAF V600E mutation, and ALK/ROS1/RET fusion. This grouping was based on the distinct biological implications of these alterations. Non-driver alterations in group B primarily affect genome stability, cell-cycle regulation, and epigenetic control, potentially increasing genomic instability and clonal heterogeneity and thereby enhancing sensitivity to cytotoxic chemotherapy. In contrast, driver alterations in group C activate bypass oncogenic signaling pathways that may confer primary resistance to EGFR-TKI inhibition, for which the addition of chemotherapy alone may be insufficient. Group A, defined by the absence of other clinically meaningful alterations, represents tumors with relatively low genomic complexity that are highly dependent on EGFR signaling. Co-mutation burden was defined as the total number of clinically meaningful gene alterations detected in addition to the EGFR-sensitizing mutation. NGS was performed using clinically validated hybrid-capture targeted panels covering 168–520 cancer-related genes (depending on the testing platform and time period), with consistent coverage of all genes relevant to the co-alteration classification in this study. For tissue samples, genomic DNA was extracted from formalin-fixed paraffin-embedded (FFPE) tumor specimens, and libraries were sequenced to a mean depth of ≥500×. For plasma samples, circulating tumor DNA (ctDNA) was extracted from peripheral blood, and libraries were sequenced to a mean depth of ≥3,000×. Bioinformatic analysis was performed using validated pipelines. Single-nucleotide variants (SNVs) and small insertions/deletions (indels) were called at a mutant allele frequency (MAF) threshold of ≥1% for tissue and ≥0.5% for plasma, with a minimum of 10 supporting reads. Copy-number variations (CNVs) were determined using normalized read-depth analysis; gene amplification was defined as a copy number (CN) ≥ 6 or a log2 ratio ≥1.0, with MET amplification specifically defined as CN ≥5 and ERBB2 (HER2) amplification as CN ≥6, consistent with current clinical diagnostic criteria. Gene fusions were detected by DNA-based breakpoint analysis. Interpretation of somatic variants and classification of clinical significance followed the joint guidelines of CAP, IASLC, and AMP, with reference to CSCO NSCLC guidelines. Variants of uncertain significance (VUS) were not counted as clinically meaningful alterations and did not influence subgroup assignment. Rare alterations with a frequency <1% in the cohort were aggregated as “other/rare” in the genomic landscape; those with established oncogenic significance were assigned to the appropriate subgroup, whereas VUS were excluded from classification. Accordingly, group A (EGFR mutation alone) was defined strictly by the absence of other known clinically meaningful alterations beyond the EGFR-sensitizing mutation, with VUS and rare variants of uncertain significance not precluding assignment to group A.

### 2.3 Efficacy assessment

Tumor imaging assessments were performed using contrast-enhanced chest CT, abdominal CT or ultrasonography, cranial MRI, and whole-body bone scan. For patients with baseline brain metastases, intracranial response was assessed according to RECIST version 1.1 for measurable intracranial lesions (defined as lesions ≥10 mm in longest diameter on contrast-enhanced cranial MRI). Intracranial PFS was evaluated in all patients with baseline brain metastases, including those with non-measurable intracranial disease. Cranial MRI was performed at baseline and every 6–9 weeks thereafter, synchronized with systemic imaging assessments, and the imaging schedule was comparable between treatment groups. Assessments were conducted every 6–9 weeks after treatment initiation until disease progression, death, or loss to follow-up. Efficacy was evaluated according to RECIST version 1.1 by two independent radiologists in a blinded manner, and classified as complete response (CR), partial response (PR), stable disease (SD), or progressive disease (PD). The objective response rate (ORR) was defined as the proportion of patients with CR or PR, and the disease control rate (DCR) as the proportion with CR, PR, or SD.

The primary endpoint was PFS, defined as the time from initiation of first-line treatment to the first documented disease progression or death from any cause, whichever occurred first; patients who were alive and progression-free were censored at the date of the last imaging assessment. Secondary endpoints included OS (defined as the time from treatment initiation to death from any cause), ORR, DCR, intracranial PFS (iPFS; defined as the time from treatment initiation to intracranial lesion progression or death in patients with baseline brain metastases), intracranial ORR (iORR), and safety.

### 2.4 Follow-up

All patients were managed under a standardized follow-up protocol. Follow-up was conducted via outpatient review, telephone contact, and review of inpatient medical records. Follow-up content encompassed: (1) survival status—confirmation of whether the patient was alive, with the date and cause of death recorded for deceased patients (tumor-related vs. non-tumor-related death); (2) disease progression—recording the date of first progression, site of progression (intracranial, extracranial, or both), and pattern of progression (local, indolent, or rapid); (3) adverse events—recording all adverse events during and after treatment, including hematologic toxicity, gastrointestinal reactions, skin toxicity, hepatic dysfunction, interstitial lung disease, and cardiac toxicity, graded according to the National Cancer Institute Common Terminology Criteria for Adverse Events (CTCAE) version 5.0 (21); and (4) quality of life and treatment adherence—recording changes in performance status and instances of, and reasons for, dose reduction or treatment interruption. Follow-up frequency was every 6–9 weeks during treatment, every 3 months for the first 2 years after treatment completion, every 6 months during years 3–5, and annually thereafter, until death or loss to follow-up. The last follow-up date was July 31, 2026. Loss to follow-up was defined as inability to contact the patient or family for two or more consecutive follow-up attempts without confirmed survival information. Median follow-up duration was estimated by the reverse Kaplan–Meier method.

### 2.5 Statistical analysis

Statistical analyses were performed using R software (version 4.3.0; https://www.r-project.org). Continuous variables are expressed as mean ± standard deviation or median (interquartile range), and between-group comparisons were made using the independent-samples t-test or Mann–Whitney U test. Categorical variables are expressed as frequency (percentage), and between-group comparisons were made using the chi-square test or Fisher's exact test.

To reduce selection bias and confounding in this retrospective study, 1:1 nearest-neighbor propensity score matching (PSM) was performed with a caliper width of 0.02. Propensity scores were estimated by multivariable logistic regression, incorporating age, sex, smoking history, ECOG score, EGFR mutation subtype, brain metastasis, liver metastasis, bone metastasis, co-alteration grouping, co-mutation burden, TKI type, and NGS sample type. Post-matching between-group balance was assessed using the standardized mean difference (SMD), with an SMD < 0.1 considered indicative of good balance. Survival curves were estimated by the Kaplan–Meier method, and between-group comparisons were made using the log-rank test. Hazard ratios (HRs) and 95% confidence intervals (CIs) were estimated by the Cox proportional hazards regression model. Multivariable Cox regression incorporated treatment modality, age, sex, ECOG score, EGFR subtype, brain metastasis, liver metastasis, co-alteration grouping, and TKI type; a treatment-by-co-alteration interaction term was included to test for heterogeneity of treatment effect across subgroups. Within each co-alteration subgroup, the HR and 95% CI for combination chemotherapy versus monotherapy were derived from the Cox model coefficients. The proportional hazards assumption was verified by Schoenfeld residual tests.

For patients with baseline brain metastases, the cumulative incidence of intracranial progression was estimated using the cumulative incidence function (CIF), with non-intracranial progression or death treated as competing events; between-group comparisons were made using Gray's test, and the subdistribution HR (sHR) was estimated by the Fine-Gray model (22). All statistical tests were two-sided, and a *P* value < 0.05 was considered statistically significant. The significance level for interaction tests was set at *α* = 0.10 to enhance the power to detect heterogeneity of subgroup effects. Given the exploratory nature of these subgroup interaction analyses and the number of interactions tested, no formal adjustment for multiple comparisons was applied.

## 3 Results

### 3.1 Patient baseline characteristics

Between January 2018 and December 2025, a total of 912 patients with advanced EGFR-mutant NSCLC who received first-line third-generation EGFR-TKI therapy were screened. After excluding 64 patients (42 with insufficient NGS data, 15 with missing follow-up, and 7 with concurrent other malignancies), 848 patients were ultimately enrolled, including 324 in the combination group and 524 in the monotherapy group (Figure 1). Following 1:1 propensity score matching, 285 patients remained in each group, yielding a final analytic cohort of 570 patients.

**Figure 1:**
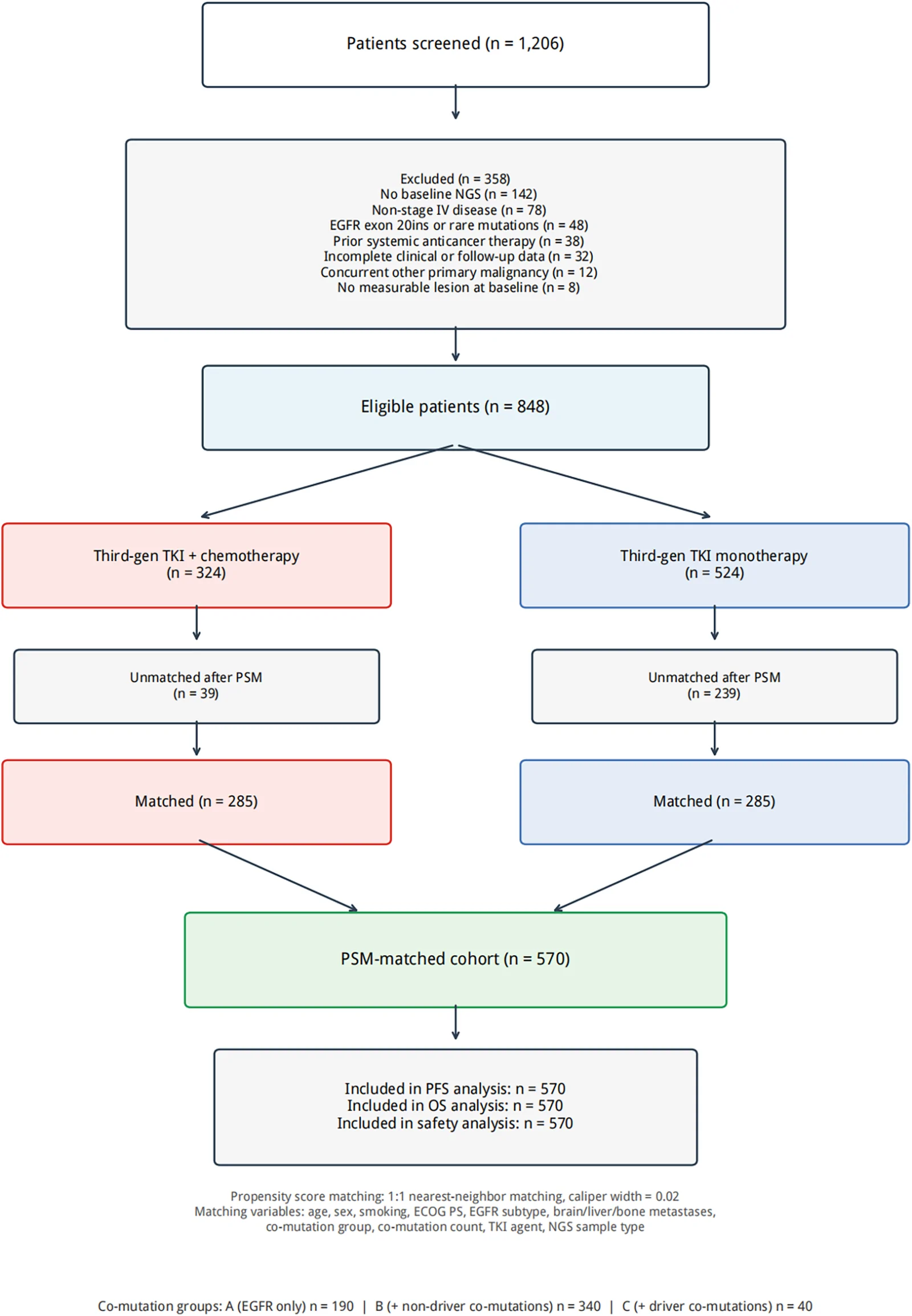
STROBE-compliant study flow diagram. Between January 2018 and December 2025, patients with advanced EGFR-mutant NSCLC who received first-line third-generation EGFR-TKI therapy were screened at two centers. Exclusion criteria included insufficient NGS data, missing follow-up, and concurrent other malignancies. A total of 848 patients were ultimately enrolled, including 324 in the combination group (TKI plus pemetrexed-platinum chemotherapy) and 524 in the monotherapy group (TKI alone). After 1:1 propensity score matching (PSM), 285 patients remained in each group (570 in total) and were included in the final analysis. Based on baseline NGS co-alteration profiles, patients were further classified into group A (EGFR mutation alone, *n* = 190), group B (concomitant non-driver co-mutations, *n* = 340), and group C (concomitant driver co-mutations, *n* = 40).

Before PSM, patients in the combination group were slightly younger than those in the monotherapy group (60.7 ± 9.8 vs. 62.3 ± 10.4 years, *P* = 0.026), and the proportion of former/current smokers was higher (36.7% vs. 29.8%, *P* = 0.035); no other statistically significant differences in baseline characteristics were observed. After PSM, the two groups achieved excellent balance across all covariates, with all SMD values below 0.035 (Table 1, Figure 3A). The matched cohort had a median age of 61.4 years, 56.5% were female, 65.8% were never smokers, 87.5% had an ECOG score of 0–1, EGFR 19del and L858R mutations accounted for 53.9% and 46.1%, respectively, and the baseline brain metastasis rate was 33.9%. Regarding co-alteration distribution after PSM, group A (EGFR mutation alone) comprised 190 patients (33.3%), group B (concomitant non-driver co-mutations) 340 patients (59.6%), and group C (concomitant driver co-mutations) 40 patients (7.0%). In the combination group, 83.5% of patients received a carboplatin-based regimen and 16.5% received cisplatin; the median number of chemotherapy cycles was 4 (IQR: 4–6), 69.5% received pemetrexed maintenance therapy, and 94.0% initiated TKI and chemotherapy synchronously. The baseline genomic co-alteration landscape is shown in Figure 2. Among the 570 post-PSM patients, TP53 was the most frequent co-mutated gene (36.8%), followed by CDKN2A/B (8.1%), PIK3CA (6.5%), RB1 (6.0%), CTNNB1 (4.6%), ARID1A (3.5%), CCND1/2/3 (3.2%), PTEN (2.5%), and STK11 (2.1%). Driver gene co-alterations were identified in 40 patients (7.0%), most commonly KRAS mutation (2.1%), MET amplification (1.8%), ERBB2 amplification or mutation (1.4%), BRAF V600E (1.1%), and ALK/ROS1/RET fusion (0.7%). An additional 19.1% of patients carried other rare alterations with a frequency below 1%. Arranged by co-alteration grouping, patients in group A had no other clinically meaningful gene alterations beyond EGFR; group B was predominantly characterized by alterations in tumor suppressor genes and pathway-related genes such as TP53, CDKN2A/B, and PIK3CA; and group C harbored well-defined oncogenic driver gene activations. The distribution of gene alteration frequencies was well balanced between the combination and monotherapy groups within each co-alteration subgroup. At the data cutoff date of July 31, 2026, the median follow-up duration, estimated by the reverse Kaplan–Meier method, was 48.0 months (IQR: 32.0–64.0). PFS events occurred in 226 patients (79.3%) in the combination group and 248 patients (87.0%) in the monotherapy group. OS events occurred in 176 patients (61.8%) in the combination group and 199 patients (69.8%) in the monotherapy group.

**Table 1:** Patient baseline characteristics before and after propensity score matching.

Characteristic	Before PSM Combination (*n* = 324)	Before PSM Monotherapy (*n* = 524)	*P* value	After PSM Combination (*n* = 285)	After PSM Monotherapy (*n* = 285)	*P* value	SMD
Age (years), mean ± SD	60.7 ± 9.8	62.3 ± 10.4	0.026	61.2 ± 9.5	61.5 ± 9.7	0.832	0.031
Sex, *n* (%)			0.054			0.735	0.028
Male	146 (45.1)	201 (38.4)		122 (42.8)	126 (44.2)		
Female	178 (54.9)	323 (61.6)		163 (57.2)	159 (55.8)		
Smoking history, *n* (%)			0.035			0.791	0.022
Never smoker	205 (63.3)	368 (70.2)		186 (65.3)	189 (66.3)		
Former/current smoker	119 (36.7)	156 (29.8)		99 (34.7)	96 (33.7)		
ECOG PS, *n* (%)			0.286			0.916	0.033
0	98 (30.2)	172 (32.8)		89 (31.2)	92 (32.3)		
1	180 (55.6)	296 (56.5)		159 (55.8)	159 (55.8)		
2	46 (14.2)	56 (10.7)		37 (13.0)	34 (11.9)		
EGFR mutation subtype, *n* (%)			0.079			0.801	0.021
Exon 19 deletion	158 (48.8)	288 (55.0)		152 (53.3)	155 (54.4)		
L858R	166 (51.2)	236 (45.0)		133 (46.7)	130 (45.6)		
Brain metastasis, *n* (%)	118 (36.4)	162 (30.9)	0.098	98 (34.4)	95 (33.3)	0.791	0.022
Liver metastasis, *n* (%)	58 (17.9)	78 (14.9)	0.245	46 (16.1)	44 (15.4)	0.818	0.020
Bone metastasis, *n* (%)	148 (45.7)	218 (41.6)	0.244	124 (43.5)	121 (42.5)	0.800	0.021
Co-alteration subgroup, *n* (%)			0.767			1.000	<0.001
Group A: EGFR mutation alone	105 (32.4)	182 (34.7)		95 (33.3)	95 (33.3)		
Group B: Non-driver co-mutations	196 (60.5)	308 (58.8)		170 (59.6)	170 (59.6)		
Group C: Driver co-mutations	23 (7.1)	34 (6.5)		20 (7.0)	20 (7.0)		
Co-mutation burden, *n* (%)			0.363			0.989	0.025
0	105 (32.4)	182 (34.7)		95 (33.3)	95 (33.3)		
1	118 (36.4)	208 (39.7)		106 (37.2)	109 (38.2)		
2	68 (21.0)	92 (17.6)		60 (21.1)	57 (20.0)		
≥ 3	33 (10.2)	42 (8.0)		24 (8.4)	24 (8.4)		
TKI type, *n* (%)			0.735			0.929	0.035
Osimertinib	168 (51.9)	286 (54.6)		152 (53.3)	148 (51.9)		
Aumolertinib	98 (30.2)	148 (28.2)		84 (29.5)	88 (30.9)		
Furmonertinib	58 (17.9)	90 (17.2)		49 (17.2)	49 (17.2)		
NGS sample type, *n* (%)			0.297			0.778	0.023
Tissue	228 (70.4)	386 (73.7)		206 (72.3)	209 (73.3)		
Plasma	96 (29.6)	138 (26.3)		79 (27.7)	76 (26.7)		
Chemotherapy regimen (combination only)							
Carboplatin, *n* (%)	-	-		238 (83.5)	-		
Cisplatin, *n* (%)	-	-		47 (16.5)	-		
No. of chemotherapy cycles, median (IQR)	-	-		4 (4–6)	-		
Pemetrexed maintenance, *n* (%)	-	-		198 (69.5)	-		
Concurrent initiation, *n* (%)	-	-		268 (94.0)	-		

Data are presented as mean ± standard deviation, median (interquartile range, IQR), or frequency (percentage). One-to-one nearest-neighbor propensity score matching (PSM) was performed with a caliper width of 0.02. The propensity score was estimated via multivariable logistic regression, incorporating age, sex, smoking history, ECOG performance status (PS), EGFR mutation subtype, brain metastasis, liver metastasis, bone metastasis, co-alteration subgroup, co-mutation burden, TKI type, and NGS sample type as covariates. SMD, standardized mean difference; ECOG, Eastern Cooperative Oncology Group; TKI, tyrosine kinase inhibitor; NGS, next-generation sequencing; IQR, interquartile range.

**Figure 2:**
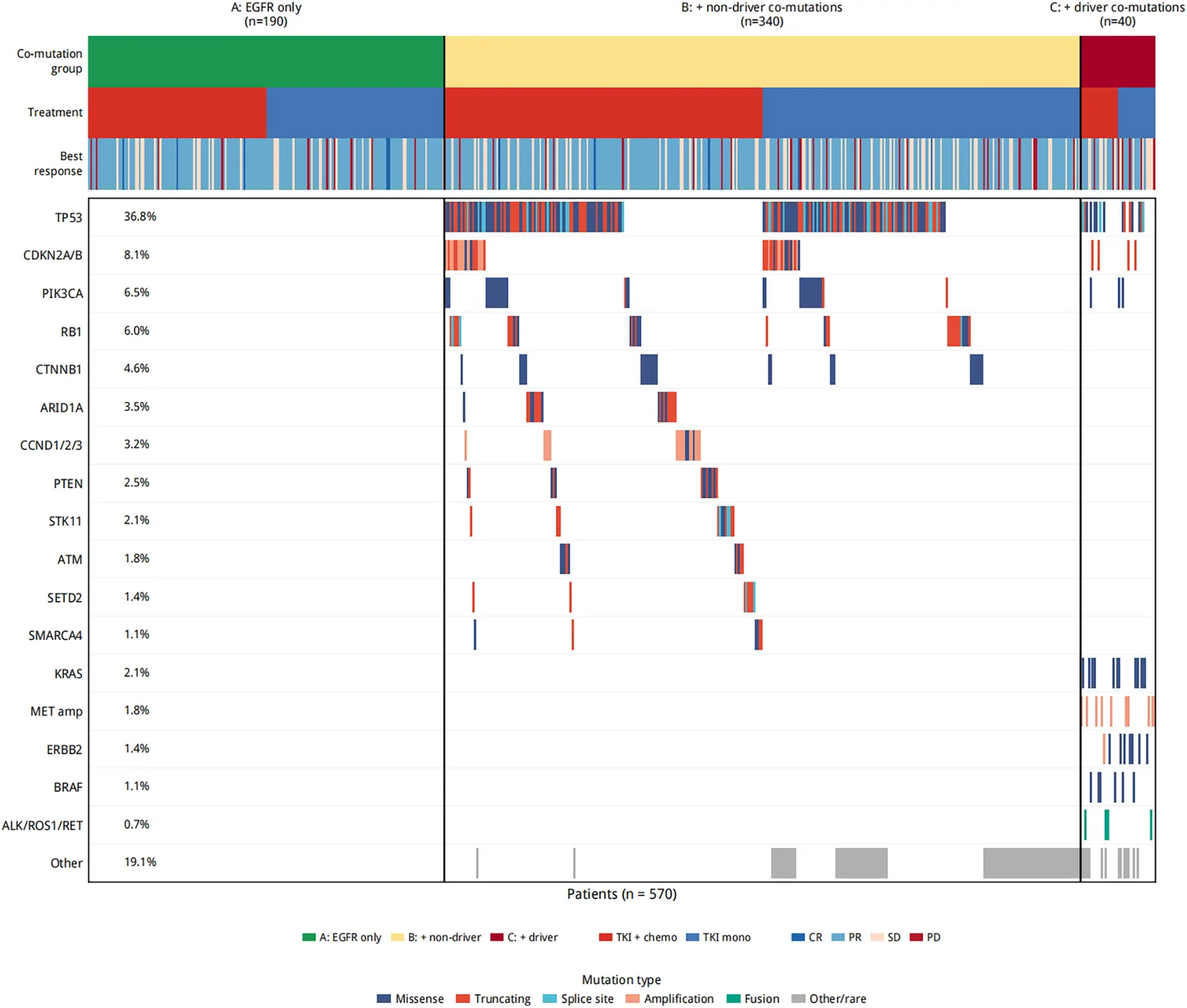
Baseline genomic co-alteration landscape (oncoprint, *n* = 570, post-PSM). Each column represents one patient, ordered by co-alteration grouping (A: EGFR mutation alone; B: concomitant non-driver co-mutations; C: concomitant driver co-mutations) and by co-mutation burden within each group. The top annotation tracks display co-alteration grouping, treatment modality (combination vs. monotherapy), and best overall response (CR/PR/SD/PD). Each row represents one gene, and colors indicate alteration type. Gene alteration frequencies are annotated on the right. ‘Other/rare’ denotes gene alterations with a cohort frequency <1% that were not displayed individually in the panel; variants of uncertain significance (VUS) were excluded from the co-alteration classification and are not shown. Among the 570 patients, TP53 was the most frequently co-altered gene (36.8%), followed by CDKN2A/B (8.1%), PIK3CA (6.5%), RB1 (6.0%), and others; driver gene co-alterations were identified in 40 patients (7.0%).

### 3.2 Overall efficacy

In the overall post-PSM cohort, the ORR was significantly higher in the combination group than in the monotherapy group (77.2% vs. 67.7%, *χ*^2^ = 6.408, *P* = 0.011), and the DCR was numerically higher but did not reach statistical significance (94.4% vs. 90.5%, *P* = 0.081) (Table 2). Survival analysis demonstrated that the median PFS was 23.3 months (95% CI: 20.9–26.2) in the combination group, numerically longer than 17.3 months (95% CI: 15.3–18.9) in the monotherapy group, corresponding to a 34.6% reduction in the risk of disease progression or death (HR = 0.654, 95% CI: 0.545–0.785, *P* < 0.001) (Figure 3B). The 1-year and 2-year PFS rates (derived from Kaplan–Meier estimates) were 77.4% and 48.7% in the combination group, respectively, compared with 65.8% and 28.7% in the monotherapy group. The median OS was 41.2 months (95% CI: 36.4–46.9) in the combination group and 33.7 months (95% CI: 29.4–40.1) in the monotherapy group, representing a 25.0% reduction in the risk of death (HR = 0.750, 95% CI: 0.612–0.919, *P* = 0.005) (Figure 3C). The 1-year, 2-year, and 3-year OS rates (derived from Kaplan–Meier estimates) were 91.9%, 73.8%, and 56.4% in the combination group, respectively, compared with 88.9%, 66.7%, and 46.3% in the monotherapy group. All landmark survival rates were extracted directly from the corresponding Kaplan–Meier curves and are internally consistent with the reported median survival estimates.

**Table 2:** Overall efficacy and efficacy by co-alteration subgroup after propensity score matching.

Efficacy endpoint	Combination (*n* = 285)	Monotherapy (*n* = 285)	HR (95% CI)	*P* value
Overall cohort				
ORR, *n* (%)	220 (77.2)	193 (67.7)	-	0.011
DCR, *n* (%)	269 (94.4)	258 (90.5)	-	0.081
Median PFS, months (95% CI)	23.3 (20.9–26.2)	17.3 (15.3–18.9)	0.654 (0.545–0.785)	<0.001
1-year PFS rate, %	77.4	65.8	-	-
2-year PFS rate, %	48.7	28.7	-	-
Median OS, months (95% CI)	41.2 (36.4–46.9)	33.7 (29.4–40.1)	0.750 (0.612–0.919)	0.005
1-year OS rate, %	91.9	88.9	-	-
2-year OS rate, %	73.8	66.7	-	-
3-year OS rate, %	56.4	46.3	-	-
Group A: EGFR mutation alone (*n* = 95 per arm)				
ORR, *n* (%)	76 (80.0)	73 (76.8)	-	0.597
DCR, *n* (%)	91 (95.8)	89 (93.7)	-	0.516
Median PFS, months (95% CI)	26.5 (21.2–29.1)	22.8 (18.7–27.2)	0.833 (0.606–1.145)	0.253
Median OS, months (95% CI)	46.7 (39.6–48.2)	44.0 (33.0–45.2)	0.841 (0.596–1.186)	0.318
Group B: Non-driver co-mutations (*n* = 170 per arm)				
ORR, *n* (%)	133 (78.2)	112 (65.9)	-	0.011
DCR, *n* (%)	161 (94.7)	154 (90.6)	-	0.146
Median PFS, months (95% CI)	23.0 (20.1–26.2)	15.3 (13.2–17.5)	0.554 (0.437–0.704)	<0.001
Median OS, months (95% CI)	39.9 (33.1–47.3)	33.1 (29.1–36.8)	0.694 (0.529–0.910)	0.007
Group C: Driver co-mutations (*n* = 20 per arm)				
ORR, *n* (%)	11 (55.0)	8 (40.0)	-	0.342
DCR, *n* (%)	17 (85.0)	15 (75.0)	-	0.695
Median PFS, months (95% CI)	14.5 (7.6–23.3)	10.2 (5.3–15.3)	0.584 (0.300–1.133)	0.088
Median OS, months (95% CI)	28.4 (13.1–34.6)	22.6 (11.8–25.3)	0.766 (0.387–1.516)	0.439
Interaction analysis				
PFS interaction *P* value	-	-	-	0.032
OS interaction *P* value	-	-	-	0.156

ORR was defined as the proportion of patients with complete response (CR) or partial response (PR); DCR was defined as the proportion with CR, PR, or stable disease (SD), assessed per RECIST version 1.1. PFS was defined as the time from initiation of first-line treatment to the first documented disease progression or death from any cause; OS was defined as the time from treatment initiation to death from any cause. Survival curves were estimated by the Kaplan–Meier method, and between-group comparisons were made using the log-rank test. Hazard ratios (HRs) and 95% confidence intervals (CIs) were calculated via Cox proportional hazards regression. Interaction *P* values were derived from Cox models including treatment × subgroup interaction terms. ORR, objective response rate; DCR, disease control rate; PFS, progression-free survival; OS, overall survival.

**Figure 3:**
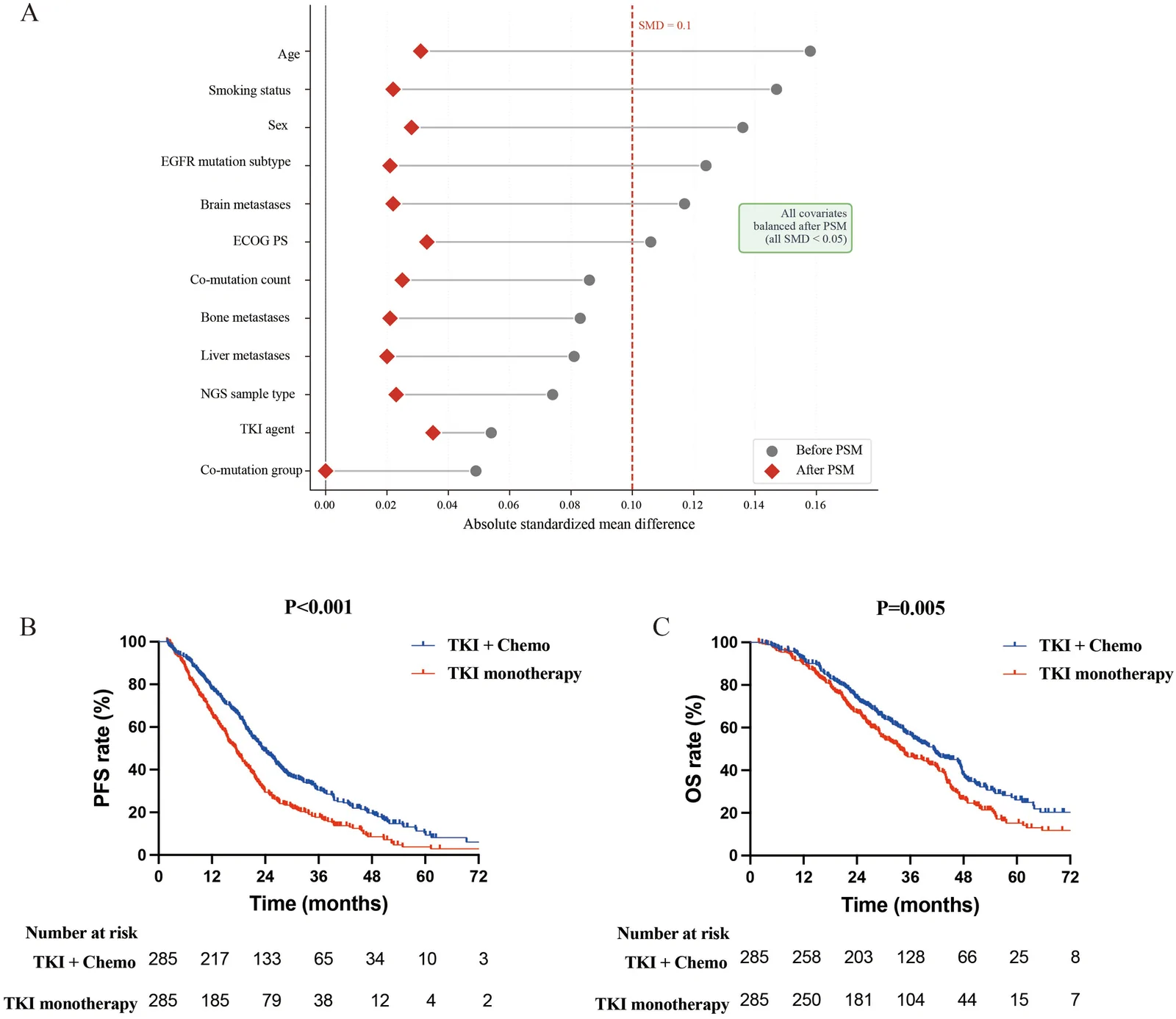
Covariate balance after PSM and overall survival analysis. **(A)** Love plot showing the absolute standardized mean difference (SMD) for all covariates before and after PSM. After matching, all covariates had SMD values below 0.035, indicating excellent between-group balance. **(B)** Kaplan–Meier curve for PFS in the overall post-PSM cohort. Median PFS was 23.3 months (95% CI: 20.9–26.2) in the combination group versus 17.3 months (95% CI: 15.3–18.9) in the monotherapy group; HR = 0.654 (95% CI: 0.545–0.785), *P* < 0.001 (log-rank test). **(C)** Kaplan–Meier curve for OS in the overall post-PSM cohort. Median OS was 41.2 months (95% CI: 36.4–46.9) in the combination group versus 33.7 months (95% CI: 29.4–40.1) in the monotherapy group; HR = 0.750 (95% CI: 0.612–0.919), *P* = 0.005.

### 3.3 Efficacy by co-alteration subgroup

In group A (EGFR mutation alone; 95 patients per arm), there were no statistically significant between-group differences in ORR (80.0% vs. 76.8%, *P* = 0.597) or DCR (95.8% vs. 93.7%, *P* = 0.516). Survival analysis showed that the median PFS was 26.5 months (95% CI: 21.2–29.1) in the combination group and 22.8 months (95% CI: 18.7–27.2) in the monotherapy group, a difference that did not reach statistical significance (HR = 0.833, 95% CI: 0.606–1.145, *P* = 0.253) (Figure 4A). The median OS was 46.7 months (95% CI: 39.6–48.2) and 44.0 months (95% CI: 33.0–45.2), respectively, also without statistical significance (HR = 0.841, 95% CI: 0.596–1.186, *P* = 0.318) (Figure 4B). In group B (concomitant non-driver co-mutations; 170 patients per arm), the ORR was significantly higher in the combination group than in the monotherapy group (78.2% vs. 65.9%, *P* = 0.011), and the DCR was 94.7% and 90.6%, respectively (*P* = 0.146). The median PFS was 23.0 months (95% CI: 20.1–26.2) in the combination group versus 15.3 months (95% CI: 13.2–17.5) in the monotherapy group, representing a 44.6% reduction in the risk of disease progression or death (HR = 0.554, 95% CI: 0.437–0.704, *P* < 0.001) (Figure 4C). The median OS was 39.9 months (95% CI: 33.1–47.3) and 33.1 months (95% CI: 29.1–36.8), respectively, corresponding to a 30.6% reduction in the risk of death (HR = 0.694, 95% CI: 0.529–0.910, *P* = 0.007) (Figure 4D). In group C (concomitant driver co-mutations; 20 patients per arm), the ORR was numerically higher in the combination group (55.0% vs. 40.0%, *P* = 0.342) but did not reach statistical significance; the DCR was 85.0% and 75.0%, respectively (Fisher's exact test, *P* = 0.695). The median PFS was 14.5 months (95% CI: 7.6–23.3) in the combination group versus 10.2 months (95% CI: 5.3–15.3) in the monotherapy group (HR = 0.584, 95% CI: 0.300–1.133, *P* = 0.088) (Figure 4E). The median OS was 28.4 months (95% CI: 13.1–34.6) and 22.6 months (95% CI: 11.8–25.3), respectively, without statistical significance (HR = 0.766, 95% CI: 0.387–1.516, *P* = 0.439) (Figure 4F). Of note, patients in group C had significantly worse overall prognosis than those in groups A and B. Given the small sample size (20 patients per arm) and the aggregation of distinct driver alterations, the efficacy estimates in group C should be interpreted as exploratory and hypothesis-generating only. Notably, the point estimates consistently favored combination therapy (PFS HR = 0.584, OS HR = 0.766), and the absence of statistical significance should not be interpreted as evidence of absent benefit, but rather as a reflection of limited statistical power and wide confidence intervals in this small subgroup.

**Figure 4:**
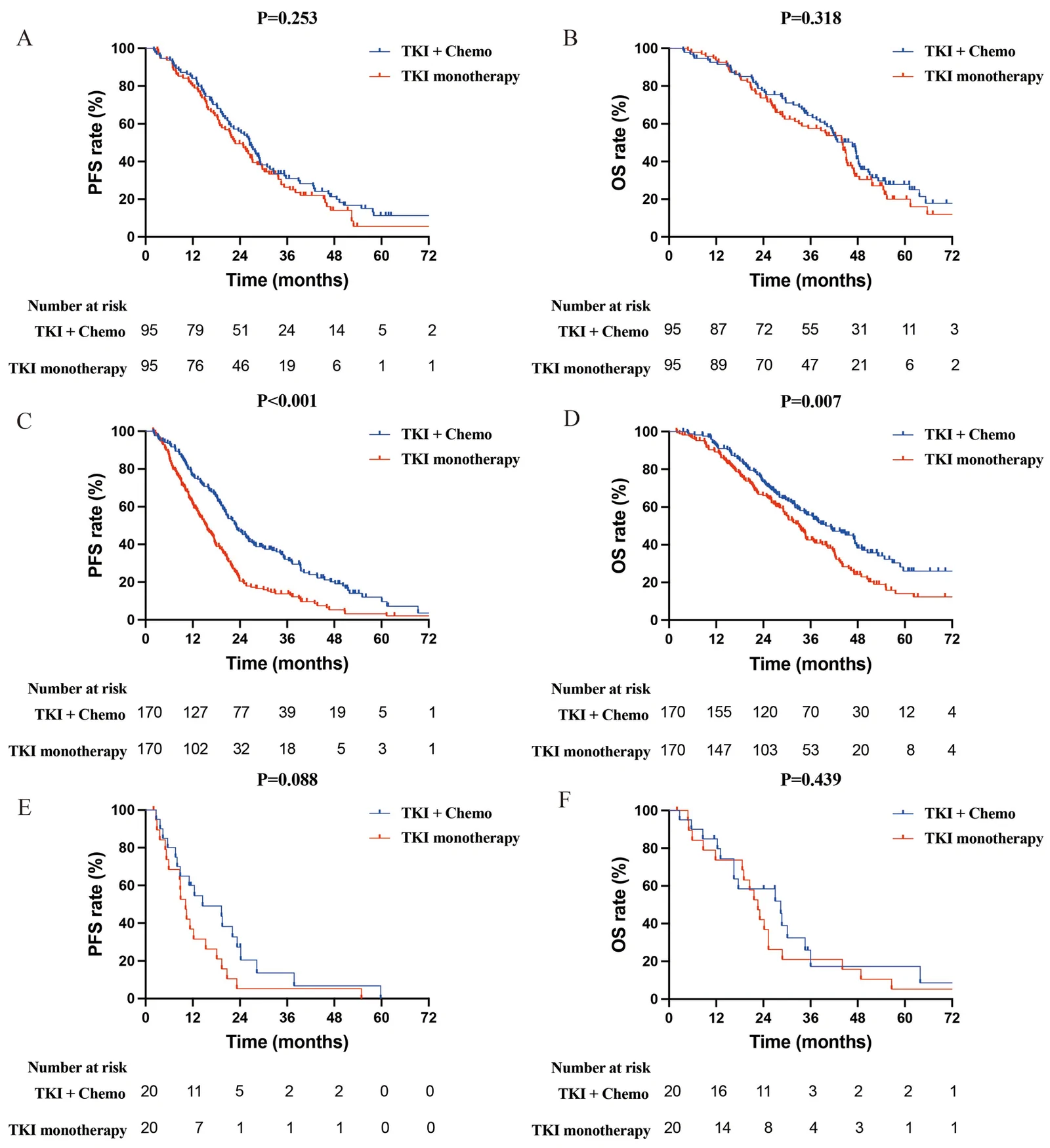
PFS and OS kaplan–meier curves by co-alteration subgroup. **(A)** PFS in group A (EGFR mutation alone; 95 patients per arm): median PFS 26.5 months (95% CI: 21.2–29.1) vs. 22.8 months (95% CI: 18.7–27.2); HR = 0.833 (95% CI: 0.606–1.145), *P* = 0.253. **(B)** OS in group A: median OS 46.7 months (95% CI: 39.6–48.2) vs. 44.0 months (95% CI: 33.0–45.2); HR = 0.841 (95% CI: 0.596–1.186), *P* = 0.318. **(C)** PFS in group B (concomitant non-driver co-mutations; 170 patients per arm): median PFS 23.0 months (95% CI: 20.1–26.2) vs. 15.3 months (95% CI: 13.2–17.5); HR = 0.554 (95% CI: 0.437–0.704), *P* < 0.001. **(D)** OS in group B: median OS 39.9 months (95% CI: 33.1–47.3) vs. 33.1 months (95% CI: 29.1–36.8); HR = 0.694 (95% CI: 0.529–0.910), *P* = 0.007. **(E)** PFS in group C (concomitant driver co-mutations; 20 patients per arm): median PFS 14.5 months (95% CI: 7.6–23.3) vs. 10.2 months (95% CI: 5.3–15.3); HR = 0.584 (95% CI: 0.300–1.133), *P* = 0.088. **(F)** OS in group C: median OS 28.4 months (95% CI: 13.1–34.6) vs. 22.6 months (95% CI: 11.8–25.3); HR = 0.766 (95% CI: 0.387–1.516), *P* = 0.439.

Interaction analysis between treatment and co-alteration grouping demonstrated a statistically significant interaction for PFS (interaction *P* = 0.032). Specifically, the treatment × group B interaction term indicated enhanced PFS benefit in group B relative to group A (HR = 0.63, 95% CI: 0.42–0.95, *P* = 0.028), whereas the treatment × group C interaction was not significant (HR = 0.71, 95% CI: 0.34–1.48, *P* = 0.36) (Tables 3). For OS, however, the interaction did not reach statistical significance (interaction *P* = 0.156; treatment × group B: HR = 0.77, 95% CI: 0.48–1.24, *P* = 0.28; treatment × group C: HR = 0.82, 95% CI: 0.36–1.86, *P* = 0.64), indicating that the differential treatment effect across subgroups was not consistently observed for overall survival. Given the exploratory nature of these interaction analyses and the absence of adjustment for multiple comparisons, these findings should be interpreted as hypothesis-generating.

**Table 3:** Multivariable cox regression analysis for PFS and OS (post-PSM cohort, *n* = 570).

Variable	PFS HR (95% CI)	PFS *P* value	OS HR (95% CI)	OS *P* value
Treatment (combination vs. monotherapy)^a^	0.82 (0.60–1.12)	0.21	0.88 (0.63–1.22)	0.43
Age (≥65 vs. < 65 years)	1.12 (0.92–1.37)	0.25	1.18 (0.92–1.52)	0.18
Sex (male vs. female)	1.08 (0.88–1.32)	0.45	1.10 (0.86–1.40)	0.45
ECOG PS (2 vs. 0–1)	1.42 (1.08–1.86)	0.012	1.55 (1.12–2.14)	0.008
EGFR subtype (L858R vs. 19del)	1.22 (1.00–1.48)	0.045	1.16 (0.92–1.46)	0.21
Brain metastasis (yes vs. no)	1.38 (1.12–1.70)	0.003	1.45 (1.12–1.88)	0.005
Liver metastasis (yes vs. no)	1.48 (1.16–1.88)	0.001	1.58 (1.15–2.17)	0.005
Co-alteration subgroup (reference: Group A)				
Group B vs. Group A	1.68 (1.24–2.28)	<0.001	1.52 (1.08–2.14)	0.016
Group C vs. Group A	2.65 (1.58–4.45)	<0.001	2.18 (1.28–3.72)	0.004
TKI type (reference: osimertinib)				
Aumolertinib vs. osimertinib	0.96 (0.78–1.18)	0.70	0.94 (0.72–1.22)	0.63
Furmonertinib vs. osimertinib	0.92 (0.72–1.18)	0.52	0.88 (0.64–1.20)	0.42
Interaction terms				
Treatment × Group B	0.63 (0.42–0.95)	0.028	0.77 (0.48–1.24)	0.28
Treatment × Group C	0.71 (0.34–1.48)	0.36	0.82 (0.36–1.86)	0.64

HR, hazard ratio; CI, confidence interval; PFS, progression-free survival; OS, overall survival; ECOG PS, Eastern Cooperative Oncology Group performance status.

a Because the model incorporates treatment × co-alteration subgroup interaction terms, the main treatment effect represents the effect of combination therapy relative to monotherapy within Group A (EGFR mutation alone). The proportional hazards assumption was verified via Schoenfeld residual tests; all variables satisfied the assumption (all *P* > 0.05).

### 3.4 Multivariable cox regression analysis

The results of multivariable Cox regression analysis are shown in Table 3. After adjusting for age, sex, ECOG score, EGFR subtype, brain metastasis, liver metastasis, co-alteration grouping, and TKI type, the treatment-by-co-alteration interaction term was significantly associated with PFS (treatment × group B: HR = 0.63, 95% CI: 0.42–0.95, *P* = 0.028). The HRs for combination chemotherapy versus monotherapy within each group, derived from the model coefficients, were as follows (Table 4): group A, PFS HR = 0.82 (95% CI: 0.60–1.12, *P* = 0.21); group B, PFS HR = 0.52 (95% CI: 0.39–0.69, *P* < 0.001); group C, PFS HR = 0.58 (95% CI: 0.28–1.20, *P* = 0.14), consistent with the Kaplan–Meier analyses. Furthermore, ECOG score of 2 (PFS HR = 1.42, *P* = 0.012; OS HR = 1.55, *P* = 0.008), brain metastasis (PFS HR = 1.38, *P* = 0.003; OS HR = 1.45, *P* = 0.005), and liver metastasis (PFS HR = 1.48, *P* = 0.001; OS HR = 1.58, *P* = 0.005) were independent adverse prognostic factors for both PFS and OS. EGFR L858R mutation subtype (compared with 19del) was an independent adverse prognostic factor for PFS (HR = 1.22, 95% CI: 1.00–1.48, *P* = 0.045) but did not significantly affect OS (HR = 1.16, *P* = 0.21). Co-alteration grouping also carried independent prognostic value: compared with group A, patients in group B (PFS HR = 1.68, *P* < 0.001; OS HR = 1.52, *P* = 0.016) and group C (PFS HR = 2.65, *P* < 0.001; OS HR = 2.18, *P* = 0.004) had significantly worse prognosis. TKI type (osimertinib vs. aumolertinib vs. furmonertinib) had no significant effect on either PFS or OS.

**Table 4:** HR and 95% CI for combination therapy vs. monotherapy within each co-alteration subgroup, derived from multivariable Cox model coefficients.

**Co-alteration subgroup**	**PFS HR (95% CI)**	**PFS *P* value**	**OS HR (95% CI)**	**OS *P* value**
Group A (*n* = 190)	0.82 (0.60–1.12)	0.21	0.88 (0.63–1.22)	0.43
Group B (*n* = 340)	0.52 (0.39–0.69)	<0.001	0.68 (0.49–0.94)	0.020
Group C (*n* = 40)	0.58 (0.28–1.20)	0.14	0.72 (0.32–1.62)	0.43

Subgroup-specific HRs were derived by combining the main treatment coefficient with the corresponding treatment   ×  subgroup interaction coefficient from the multivariable Cox model presented in Table 3. Group A serves as the reference subgroup for the interaction term.

The PFS subgroup analysis is presented in Figure 5. The PFS benefit of combined chemotherapy was consistent in direction across all prespecified subgroups (all HRs < 1), including age, sex, smoking history, ECOG PS, EGFR mutation subtype, brain metastasis, liver metastasis, NGS sample type, and TKI type. Interaction tests showed that only the interaction between co-alteration grouping and treatment modality was statistically significant (*P* interaction = 0.032), whereas all other subgroup *P* interactions were > 0.05. Among the co-alteration groups, group B (concomitant non-driver co-mutations) derived the most pronounced benefit (HR = 0.52, 95% CI: 0.39–0.69), group A (EGFR mutation alone) did not achieve statistically significant benefit (HR = 0.82, 95% CI: 0.60–1.12), and group C, with a small sample size (20 per arm), had an HR of 0.58 (95% CI: 0.28–1.20).

**Figure 5:**
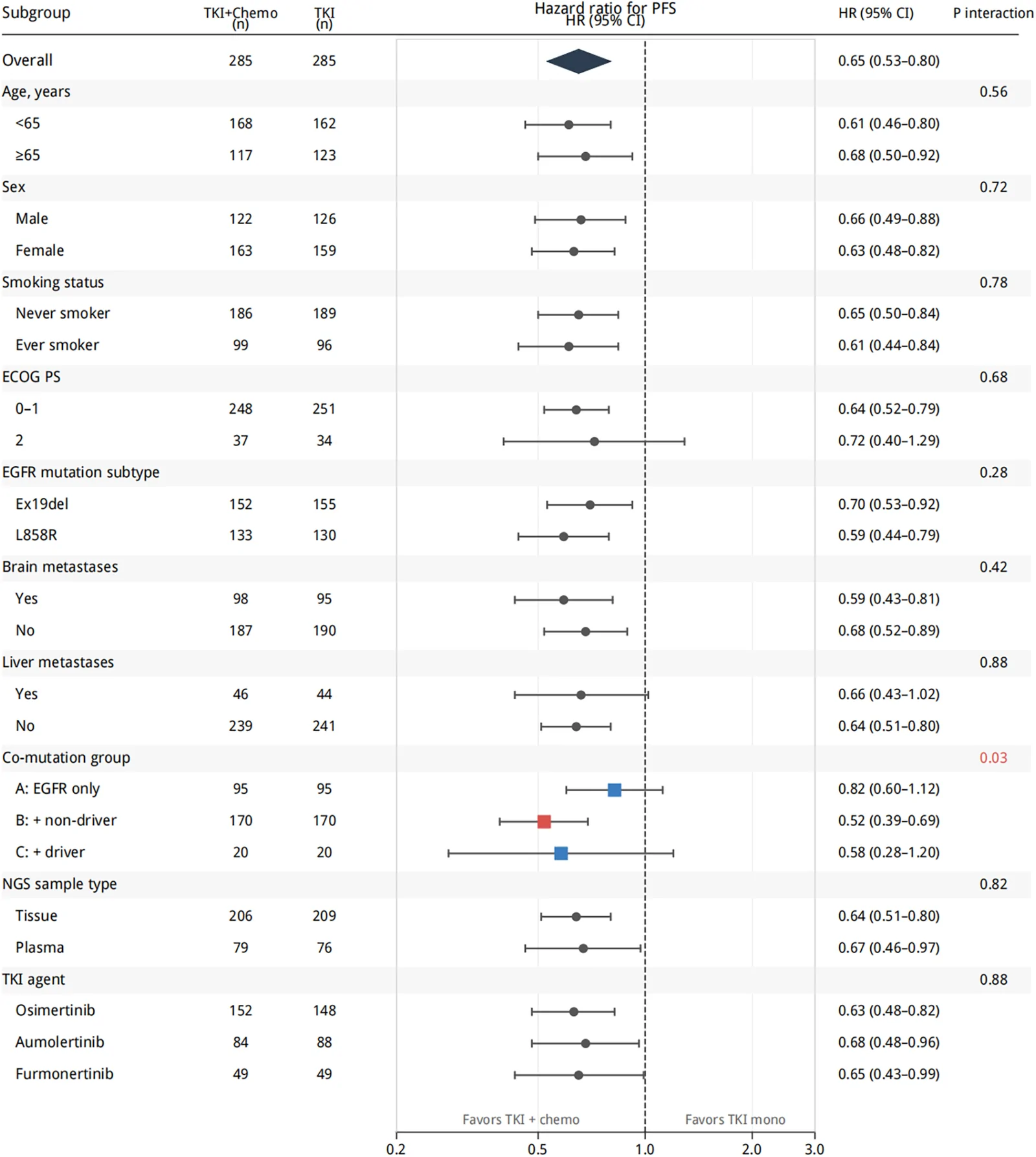
Forest plot of PFS subgroup analysis. Hazard ratios (HRs) and 95% confidence intervals for PFS with combination chemotherapy versus monotherapy across prespecified subgroups in the post-PSM cohort. Subgroups include age, sex, smoking history, ECOG performance status score, EGFR mutation subtype, brain metastasis, liver metastasis, NGS sample type, TKI type, and co-alteration grouping. Interaction *P* values were derived from the treatment-by-subgroup interaction term in the Cox model. The interaction between co-alteration grouping and treatment modality was statistically significant (*P* interaction = 0.032). Group B (concomitant non-driver co-mutations) derived the most pronounced benefit (HR = 0.52, 95% CI: 0.39–0.69), whereas group A (EGFR mutation alone) did not achieve statistically significant benefit (HR = 0.82, 95% CI: 0.60–1.12).

### 3.5 Association between co-mutation burden and efficacy

To further explore the impact of co-mutation burden on the benefit of combined chemotherapy, we performed stratified analyses by the number of co-mutations (Table 5). The results showed that, as the number of co-mutations increased, the median PFS in the monotherapy group declined progressively (0 mutations: 22.8 months; 1 mutation: 18.6 months; 2 mutations: 13.5 months; ≥3 mutations: 11.5 months), whereas the combination group demonstrated PFS benefit across all strata. Specifically, the benefit of combined chemotherapy was statistically significant in patients with 1 co-mutation (HR = 0.594, *P* < 0.001) (Figure 6A), 2 co-mutations (HR = 0.474, *P* < 0.001) (Figure 6B), and ≥3 co-mutations (HR = 0.519, *P* = 0.015) (Figure 6C), whereas the benefit in patients with 0 co-mutations (i.e., group A) did not reach significance (HR = 0.833, *P* = 0.253). The test for trend in the interaction between co-mutation number and treatment modality for PFS yielded *P* = 0.021, suggesting that patients with higher co-mutation burden may derive greater benefit from combined chemotherapy (Figure 6D).

**Table 5:** Progression-free survival analysis stratified by co-mutation burden.

**Co-mutation burden**	**Combination *n* (%)**	**Monotherapy *n* (%)**	**Combination median PFS, months (95% CI)**	**Monotherapy median PFS, months (95% CI)**	**HR (95% CI)**	***P*** **value**
0	95 (33.3)	95 (33.3)	26.5 (21.2–29.1)	22.8 (18.7–27.2)	0.833 (0.606–1.145)	0.253
1	106 (37.2)	109 (38.2)	25.5 (21.1–30.1)	18.6 (14.7–20.7)	0.594 (0.447–0.789)	<0.001
2	60 (21.1)	57 (20.0)	21.3 (13.9–33.8)	13.5 (9.1–14.8)	0.474 (0.317–0.710)	<0.001
≥3	24 (8.4)	24 (8.4)	17.7 (6.7–34.7)	11.5 (6.6–16.3)	0.519 (0.282–0.955)	0.015
*P* for trend						0.021

Co-mutation burden was defined as the total number of clinically meaningful gene alterations detected in addition to the EGFR-sensitizing mutation, as determined by baseline next-generation sequencing. PFS within each stratum was estimated by the Kaplan–Meier method, with between-group comparisons using the log-rank test. HRs and 95% CIs were estimated via Cox proportional hazards regression. *P* for trend was obtained by including co-mutation burden as an ordinal variable in the Cox model and testing the significance of the coefficient. PFS, progression-free survival; HR, hazard ratio; CI, confidence interval.

**Figure 6:**
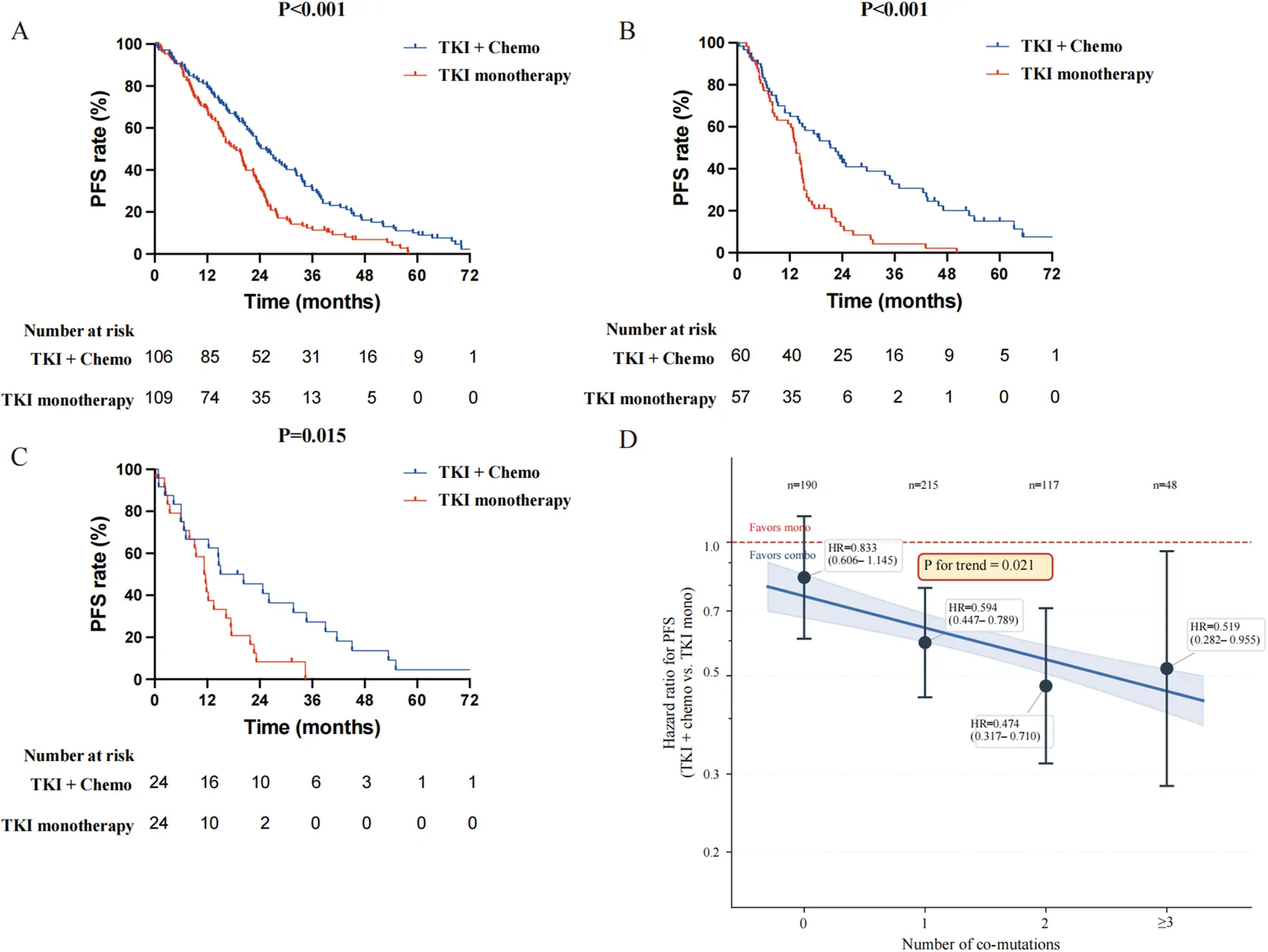
Association between co-mutation burden and PFS benefit. **(A)** Kaplan–Meier PFS curve for patients with 1 co-mutation (combination *n* = 106, monotherapy *n* = 109): HR = 0.594 (95% CI: 0.447–0.789), *P* < 0.001. **(B)** PFS curve for patients with 2 co-mutations (combination *n* = 60, monotherapy *n* = 57): HR = 0.474 (95% CI: 0.317–0.710), *P* < 0.001. **(C)** PFS curve for patients with ≥3 co-mutations (combination *n* = 24, monotherapy *n* = 24): HR = 0.519 (95% CI: 0.282–0.955), *P* = 0.015. **(D)** Trend plot of the interaction between co-mutation burden and PFS benefit from combination chemotherapy. The *x*-axis represents co-mutation burden (0, 1, 2, ≥3), and the *y*-axis represents the PFS HR and 95% CI for combination chemotherapy versus monotherapy. As co-mutation burden increased, the PFS benefit from combination chemotherapy showed an increasing trend (*P* for interaction trend = 0.021).

### 3.6 Intracranial efficacy

In the post-PSM cohort, a total of 193 patients had baseline brain metastases (98 in the combination group, 95 in the monotherapy group). The iORR was numerically higher in the combination group (63.3% vs. 50.5%, *P* = 0.074), and the iDCR was 89.8% and 82.1%, respectively (*P* = 0.124) (Table 6). The median iPFS was 18.8 months (95% CI: 15.0–21.3) in the combination group, numerically longer than 12.6 months (95% CI: 10.2–15.2) in the monotherapy group, representing a 26.1% reduction in the risk of intracranial progression or death (HR = 0.739, 95% CI: 0.544–1.003, log-rank *P* = 0.047) (Figure 7A). Given the borderline statistical significance and the upper limit of the 95% CI approaching 1.0, these intracranial efficacy findings should be interpreted cautiously. The proportion of patients with intracranial progression as the first progression event was significantly lower in the combination group (18.4% vs. 33.7%, *χ*^2^ = 5.896, *P* = 0.015).

**Table 6:** Intracranial efficacy analysis in patients with baseline brain metastases (post-PSM, *n* = 193).

Intracranial efficacy endpoint	Combination (*n* = 98)	Monotherapy (*n* = 95)	HR/sHR (95% CI)	*P* value
iORR, *n* (%)	62 (63.3)	48 (50.5)	*χ*^2^ = 3.194	0.074
iDCR, *n* (%)	88 (89.8)	78 (82.1)	χ^2^ = 2.371	0.124
Median iPFS, months (95% CI)	18.8 (15.0–21.3)	12.6 (10.2–15.2)	0.739 (0.544–1.003)	0.047
Intracranial progression as first progression, *n* (%)	18 (18.4)	32 (33.7)	χ^2^ = 5.896	0.015
Cumulative incidence of CNS progression				
12 months, %	32.2	48.6	sHR 0.74 (0.54–1.00)	0.050
24 months, %	67.2	79.9		
Median iPFS by co-alteration subgroup, months				
Group A (combination 32/monotherapy 30)	22.5 (15.9–27.2)	19.4 (10.8–25.4)	1.026 (0.596–1.768)	0.924
Group B (combination 58/monotherapy 56)	17.8 (13.7–21.0)	11.8 (8.4–13.6)	0.656 (0.441–0.977)	0.031
Group C (combination 8/monotherapy 9)	12.4 (5.6–24.1)	10.35 (4.8–13.3)	0.516 (0.181–1.471)	0.167

Intracranial efficacy was evaluated according to RECIST version 1.1 for measurable intracranial lesions. iORR was defined as the proportion of patients with intracranial CR or PR; iDCR was defined as the proportion with intracranial CR, PR, or SD. iPFS was defined as the time from treatment initiation to intracranial lesion progression or death from any cause, estimated by the Kaplan–Meier method with between-group comparison via the log-rank test. The cumulative incidence of CNS progression was estimated using the cumulative incidence function (CIF) with death as a competing risk; sHR denotes the subdistribution hazard ratio. iORR, intracranial objective response rate; iDCR, intracranial disease control rate; iPFS, intracranial progression-free survival; CNS, central nervous system; sHR, subdistribution hazard ratio; CI, confidence interval.

**Figure 7:**
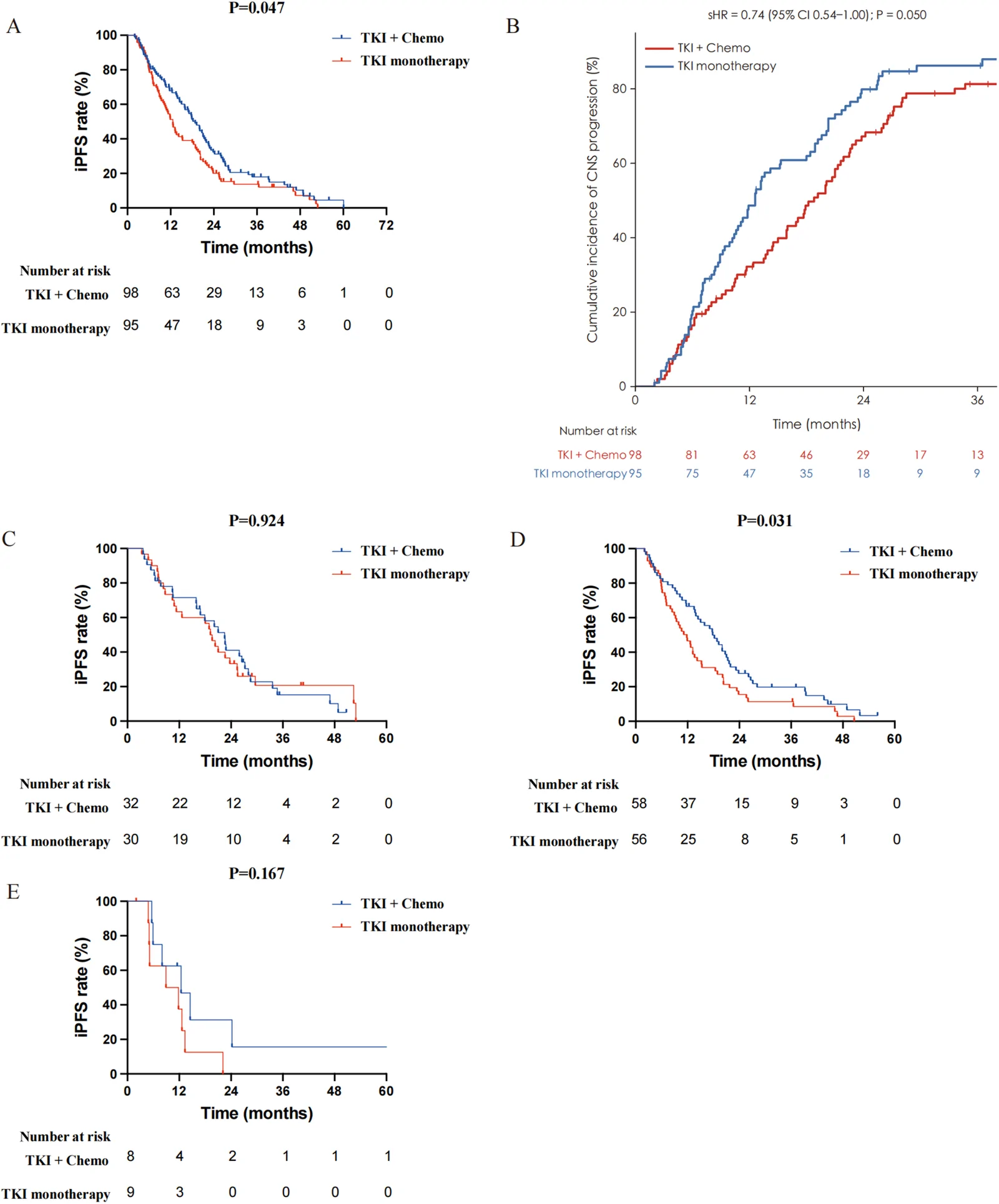
Intracranial efficacy analysis in patients with baseline brain metastases (*n* = 193). **(A)** Kaplan–Meier curve for intracranial PFS (iPFS). The combination group (*n* = 98) had a median iPFS of 18.8 months (95% CI: 15.0–21.3) versus 12.6 months (95% CI: 10.2–15.2) in the monotherapy group (*n* = 95); HR = 0.739 (95% CI: 0.544–1.003), *P* = 0.047 (log-rank test). The proportion of patients with intracranial progression as the first progression event was significantly lower in the combination group (18.4% vs. 33.7%, *χ*^2^ = 5.896, *P* = 0.015). **(B)** Cumulative incidence curve for CNS progression (competing risk analysis), treating non-CNS progression death as a competing event. 12-month CIF: 32.2% (combination) vs. 48.6% (monotherapy); 24-month: 67.2% vs. 79.9%; 36-month: 81.3% vs. 86.2%. Fine-Gray model sHR=0.74 (95% CI: 0.54–1.00), *P* = 0.050 (Gray's test). **(C)** iPFS in group A (combination *n* = 32, monotherapy *n* = 30): median iPFS 22.5 vs. 19.4 months, HR = 1.026, *P* = 0.924. **(D)** iPFS in group B (combination *n* = 58, monotherapy *n* = 56): median iPFS 17.8 vs. 11.8 months, HR = 0.656, *P* = 0.031. **(E)** iPFS in group C (combination *n* = 8, monotherapy *n* = 9): median iPFS 12.4 vs. 10.35 months, HR = 0.516, *P* = 0.167.

Competing risk analysis showed that the cumulative incidence of CNS progression at 12, 24, and 36 months was 32.2%, 67.2%, and 81.3% in the combination group, respectively, compared with 48.6%, 79.9%, and 86.2% in the monotherapy group (Figure 7B). The sHR estimated by the Fine-Gray model was 0.74 (95% CI: 0.54–1.00, *P* = 0.050). Subgroup analysis of iPFS by co-alteration grouping showed that patients in group B derived the most pronounced intracranial benefit from combined chemotherapy (m-iPFS: 17.8 vs. 11.8 months, HR = 0.656, *P* = 0.031), whereas the differences in group A (22.5 vs. 19.4 months, *P* = 0.924) and group C (12.4 vs. 10.35 months, *P* = 0.167) did not reach statistical significance (Figures 7C–E).

### 3.7 Safety

Safety analysis was based on the 570 post-PSM patients (Table 7, Figure 8). The incidence of any-grade adverse events was 100% in the combination group and 96.8% in the monotherapy group (*P* = 0.004). The incidence of grade ≥3 adverse events was significantly higher in the combination group (49.8% vs. 20.4%, *P* < 0.001). Hematologic toxicity was the most common adverse event in the combination group. The incidences of neutropenia (58.9% vs. 9.8%; grade ≥3: 25.3% vs. 1.4%), leukopenia (49.8% vs. 7.7%; grade ≥3: 16.8% vs. 0.7%), anemia (54.7% vs. 16.8%; grade ≥3: 13.3% vs. 1.8%), and thrombocytopenia (34.4% vs. 6.3%; grade ≥3: 9.8% vs. 0.4%) were all significantly elevated in the combination group (all *P* < 0.001). With respect to gastrointestinal reactions, the incidences of nausea (48.4% vs. 18.2%), vomiting (30.2% vs. 8.4%), constipation (27.4% vs. 11.2%), and decreased appetite (41.4% vs. 20.4%) were all significantly higher in the combination group, although the incidences of grade ≥3 events were low (all <5%). There was no significant between-group difference in the incidence of diarrhea (32.3% vs. 27.4%, *P* = 0.200). No significant between-group differences were observed in the incidences of rash or hepatic dysfunction (ALT and AST increased). The incidence of interstitial lung disease was low: 2.8% in the combination group (grade ≥3: 1.1%) and 2.5% in the monotherapy group (grade ≥3: 0.7%), with no significant difference (*P* = 0.794 for all grades and *P* = 1.000 for grade ≥3). The incidences of QT interval prolongation were 4.2% and 3.5%, respectively, predominantly of low grade.

**Table 7:** Summary of treatment-related adverse events (post-PSM cohort, *n* = 570).

**Adverse event**	**Combination All grades** **n (%)**	**Combination Grade ≥3** **n (%)**	**Monotherapy All grades** **n (%)**	**Monotherapy Grade ≥3** **n (%)**	***P* value (All grades)**	***P* value (Grade ≥3)**
Any adverse event	285 (100.0)	142 (49.8)	276 (96.8)	58 (20.4)	0.004	<0.001
Hematologic toxicities						
Neutropenia	168 (58.9)	72 (25.3)	28 (9.8)	4 (1.4)	<0.001	<0.001
Leukopenia	142 (49.8)	48 (16.8)	22 (7.7)	2 (0.7)	<0.001	<0.001
Anemia	156 (54.7)	38 (13.3)	48 (16.8)	5 (1.8)	<0.001	<0.001
Thrombocytopenia	98 (34.4)	28 (9.8)	18 (6.3)	1 (0.4)	<0.001	<0.001
Gastrointestinal reactions						
Nausea	138 (48.4)	12 (4.2)	52 (18.2)	2 (0.7)	<0.001	0.007
Vomiting	86 (30.2)	8 (2.8)	24 (8.4)	0	<0.001	0.007
Diarrhea	92 (32.3)	6 (2.1)	78 (27.4)	4 (1.4)	0.200	0.523
Constipation	78 (27.4)	2 (0.7)	32 (11.2)	0	<0.001	0.499
Decreased appetite	118 (41.4)	10 (3.5)	58 (20.4)	2 (0.7)	<0.001	0.020
Skin toxicity						
Rash	88 (30.9)	5 (1.8)	102 (35.8)	6 (2.1)	0.214	0.761
Hepatic dysfunction						
ALT increased	82 (28.8)	14 (4.9)	68 (23.9)	8 (2.8)	0.183	0.192
AST increased	72 (25.3)	12 (4.2)	60 (21.1)	6 (2.1)	0.233	0.151
Pulmonary toxicity						
Interstitial lung disease	8 (2.8)	3 (1.1)	7 (2.5)	2 (0.7)	0.794	1.000
Cardiac toxicity						
QT prolongation	12 (4.2)	2 (0.7)	10 (3.5)	1 (0.4)	0.664	1.000
Other						
Fatigue	128 (44.9)	8 (2.8)	72 (25.3)	3 (1.1)	<0.001	0.128
Stomatitis	58 (20.4)	3 (1.1)	42 (14.7)	1 (0.4)	0.078	0.624
Pyrexia	42 (14.7)	2 (0.7)	18 (6.3)	0	0.001	1.000
Summary						
Serious adverse events (SAEs)	—	38 (13.3)	—	18 (6.3)	—	0.005
TKI dose reduction	—	32 (11.2)	—	18 (6.3)	—	0.038
TKI discontinuation	—	22 (7.7)	—	12 (4.2)	—	0.077
Chemotherapy discontinuation	—	18 (6.3)	—	—	—	—
Treatment-related death	—	1 (0.4)^a^	—	0	—	—

Adverse events were graded and recorded according to the National Cancer Institute Common Terminology Criteria for Adverse Events (CTCAE) version 5.0. Between-group comparisons for all-grade and grade ≥3 event rates were performed using the χ^2^ test or Fisher's exact test, as appropriate. Serious adverse events (SAEs) were defined as events resulting in death, life-threatening conditions, hospitalization or prolongation of hospitalization, persistent or significant disability/functional impairment, or other medically important events.

a One patient in the combination group died from neutropenic sepsis. ALT, alanine aminotransferase; AST, aspartate aminotransferase; TKI, tyrosine kinase inhibitor; AE, adverse event.

**Figure 8:**
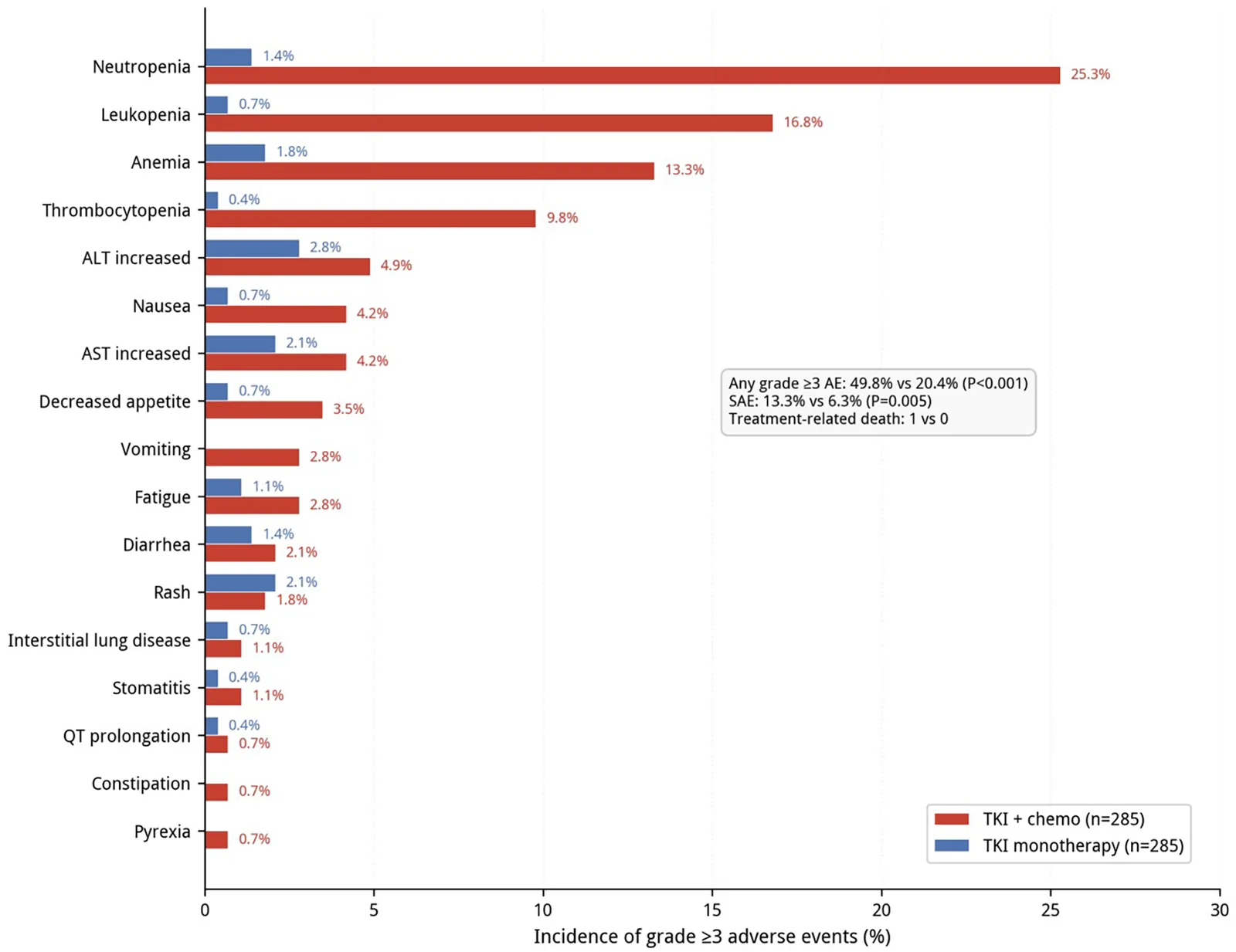
Treatment-related adverse events. Horizontal bar chart showing the incidence of treatment-related grade ≥3 adverse events in the PSM-matched cohort. Adverse events are ordered by descending incidence in the combination group. Hematologic toxicities (neutropenia, leukopenia, anemia, thrombocytopenia) were significantly elevated in the combination group, whereas the incidences of non-hematologic toxicities were generally comparable between groups. Overall, the incidence of any grade ≥3 adverse event was 49.8% versus 20.4% (*P* < 0.001), and the incidence of serious adverse events was 13.3% versus 6.3% (*P* = 0.005). One treatment-related death (neutropenic sepsis) occurred in the combination group; no treatment-related deaths occurred in the monotherapy group.

The incidence of serious adverse events (SAEs) was 13.3% in the combination group, significantly higher than 6.3% in the monotherapy group (*P* = 0.005). The proportion of patients with TKI dose reduction due to adverse events was 11.2% in the combination group, higher than 6.3% in the monotherapy group (*P* = 0.038); the TKI discontinuation rates were 7.7% and 4.2%, respectively (*P* = 0.077). One patient in the combination group died from neutropenic sepsis; no treatment-related deaths occurred in the monotherapy group.

## 4 Discussion

To the best of our knowledge, this study represents one of the largest real-world investigations to date examining the influence of genomic co-alteration patterns on the benefit of first-line third-generation EGFR-TKI plus chemotherapy in patients with advanced EGFR-mutant NSCLC. By applying propensity score matching to 848 patients and performing systematic co-alteration stratification based on baseline NGS results, we obtained several clinically meaningful findings. First, in the overall matched cohort, third-generation EGFR-TKI combined with pemetrexed-platinum chemotherapy significantly prolonged both PFS and OS compared with TKI monotherapy, which is consistent with the findings of prospective randomized controlled trials such as FLAURA2 and AENEAS2 (9, 11) and further validates the effectiveness of the combination strategy in a real-world population. Second—and most centrally—the degree of benefit from combined chemotherapy varied significantly across co-alteration backgrounds: patients with concomitant non-driver co-mutations derived the most pronounced benefit, with significant improvements in both PFS and OS; those with EGFR mutation alone achieved PFS exceeding 22 months and OS exceeding 44 months with TKI monotherapy, and the incremental benefit of adding chemotherapy was limited and non-significant; and although patients with concomitant driver co-mutations numerically benefited from combination therapy, the small sample size precluded statistical significance, and this subgroup had the worst overall prognosis. The treatment-by-co-alteration interaction for PFS was statistically significant (interaction *P* = 0.032), suggesting that co-alteration patterns may be associated with heterogeneity in treatment benefit. However, the interaction for OS was not significant (*P* = 0.156), and these retrospective findings should be regarded as hypothesis-generating rather than definitive evidence of a predictive biomarker.

These findings carry important biological and clinical implications. Tumors with EGFR mutation alone typically exhibit lower genomic complexity and clonal heterogeneity and are strongly dependent on the EGFR signaling pathway; consequently, TKI monotherapy can achieve deep and durable responses (23). In the present study, the median PFS in the monotherapy arm of group A reached 22.8 months and the median OS reached 44.0 months—results that even exceed the overall population data from the FLAURA trial (mPFS 18.9 months, mOS 38.6 months) (4, 5), possibly because group A in our study excluded co-alterations, which are themselves adverse prognostic factors. In this context, although the addition of chemotherapy numerically prolonged PFS (26.5 vs. 22.8 months), the absolute benefit was only approximately 3.7 months and was accompanied by significantly increased hematologic toxicity and SAE risk, suggesting that the net clinical benefit may be limited. These results indicate that for patients with EGFR mutation alone, TKI monotherapy may remain a reasonable first-line option, particularly for those who cannot tolerate chemotherapy or for whom quality of life is a priority. In contrast, tumors harboring non-driver co-mutations exhibit greater genomic instability and clonal diversity. TP53, the most frequently co-mutated gene, plays a central role in maintaining genomic stability, DNA damage repair, and apoptosis; its functional loss promotes the emergence and expansion of drug-resistant subclones (24, 25). Activating alterations in PIK3CA may provide parallel survival signals through the PI3 K/AKT/mTOR pathway, reducing tumor dependence on EGFR inhibition (26). Against this biological backdrop, the cytotoxic effect of chemotherapy may non-selectively eliminate rapidly proliferating tumor cells—including TKI-insensitive clones—thereby delaying the onset of resistance. In our study, the PFS HR for combined chemotherapy in group B reached 0.554, with an absolute PFS benefit of 7.7 months and an OS benefit of 6.8 months—a magnitude comparable to or even exceeding that observed in the overall FLAURA2 population—suggesting that patients in group B may derive substantial benefit from combination therapy. However, group B comprises biologically heterogeneous non-driver alterations, and TP53 was by far the most frequent co-alteration (36.8%); therefore, we cannot exclude the possibility that the observed benefit was driven primarily by TP53-mutant disease rather than by non-driver co-alterations broadly. Alteration-specific analyses would be required to clarify this question, but were not feasible in the present cohort due to limited sample sizes within individual genomic alterations. Notably, the TP53 co-mutation prevalence in our cohort (36.8%) was somewhat lower than the 50%–60% frequently reported in the literature (14, 15). Several factors may account for this difference. First, the cited estimates were derived predominantly from Western cohorts and earlier-generation sequencing panels, whereas emerging data from Asian EGFR-mutant cohorts have reported TP53 co-mutation rates ranging from 35% to 50%, suggesting potential ethnic and geographic variation. Second, approximately 27% of our patients underwent plasma rather than tissue NGS; plasma ctDNA testing may underestimate the prevalence of subclonal mutations, including TP53, particularly in patients with low tumor shedding. Third, differences in NGS panel design, variant-calling thresholds, and the exclusion of VUS from classification may further contribute to the lower observed prevalence. These potential influences should be considered when interpreting the generalizability of our co-alteration findings. Furthermore, the stratified analysis by co-mutation burden supports this interpretation: as the number of co-mutations increased from 0 to ≥3, the median PFS in the monotherapy group declined progressively from 22.8 to 11.5 months, whereas combined chemotherapy conferred significant PFS benefit in all subgroups with ≥1 co-mutation, and the test for interaction trend yielded *P* = 0.021. This dose–response relationship further supports the biological plausibility that co-alteration burden may be associated with differential benefit from combination therapy, although prospective validation is required. Patients in group C (concomitant driver co-mutations) had the worst prognosis among the three groups, with a median PFS of only 10.2 months and median OS of only 22.6 months in the monotherapy arm, consistent with prior reports that EGFR-mutant tumors with concomitant driver gene alterations respond poorly to TKI therapy (27). Although combined chemotherapy numerically prolonged PFS (14.5 vs. 10.2 months) and OS (28.4 vs. 22.6 months), the differences did not reach statistical significance. This may be attributable to two factors: first, the small sample size of this subgroup (20 patients per arm), which aggregated several distinct driver alterations, severely limited statistical power, and these results should be considered exploratory only; second, for tumors with well-defined bypass driver activation (such as MET amplification or RAS mutations), the simple addition of chemotherapy may be insufficient to overcome driver-mediated resistance. Accordingly, the optimal first-line strategy for patients in group C warrants further investigation, and future studies should explore precision combination approaches tailored to specific driver co-alterations.

With respect to intracranial efficacy, combined chemotherapy was associated with a numerically longer iPFS in patients with baseline brain metastases (18.8 vs. 12.6 months; HR = 0.739, 95% CI 0.544–1.003; log-rank *P* = 0.047), although this difference was of borderline statistical significance, with the upper limit of the 95% CI approaching 1.0, and should therefore be interpreted cautiously, consistent with the Results. Combination therapy also reduced the proportion of patients with intracranial progression as the first progression event (18.4% vs. 33.7%, *P* = 0.015). These results are consistent with the CNS analysis of the FLAURA2 trial, which demonstrated significantly superior CNS PFS with osimertinib plus chemotherapy versus monotherapy (24.9 vs. 13.8 months) (10). It is noteworthy that although pemetrexed and platinum agents have limited blood–brain barrier penetration, they may achieve effective intracranial drug concentrations when brain metastases compromise the integrity of the blood–brain barrier, thereby exerting synergistic activity with osimertinib. Subgroup analysis of iPFS by co-alteration grouping likewise showed that patients in group B derived the most pronounced intracranial benefit, consistent with the pattern observed for systemic efficacy, suggesting that the influence of co-alteration patterns is consistent across intracranial and extracranial disease. In terms of safety, the toxicity profile of combination therapy was as expected, primarily manifested as increased chemotherapy-related hematologic toxicity. The incidence of grade ≥3 neutropenia in the combination group was 25.3%, comparable to that reported in the FLAURA2 trial. Importantly, the incidence of interstitial lung disease was low and did not differ significantly between groups (2.8% vs. 2.5%), consistent with the known safety profile of third-generation EGFR-TKIs. The one treatment-related death in the combination group (from neutropenic sepsis) underscores the need for careful assessment of chemotherapy tolerance and close monitoring of complete blood counts and signs of infection when selecting combination therapy, particularly in elderly patients, those with poor performance status, or those with significant comorbidities.

Several limitations of this study should be acknowledged. First, as a non-randomized retrospective study, despite the use of PSM to balance measured baseline characteristics, residual confounding from unmeasured factors cannot be excluded. Factors including disease burden, symptomatic disease, CNS disease severity, comorbidities, chemotherapy fitness and organ function, physician and patient treatment preferences, and calendar period may have influenced the allocation to combination therapy versus monotherapy and were not fully captured in the propensity model. This limitation is particularly relevant when interpreting treatment-by-genomic subgroup differences, as imbalances in these unmeasured factors could potentially confound the observed interaction between co-alteration status and treatment modality. In particular, OS as a secondary endpoint is strongly affected by post-progression therapy. Given the long study period (2018–2025), during which therapeutic options for EGFR-mutant NSCLC evolved substantially—including the availability of novel targeted agents, antibody-drug conjugates, and immunotherapy combinations—subsequent treatment patterns may have differed between treatment groups and over calendar time. However, detailed post-progression treatment data were not systematically collected in this retrospective cohort, and the potential impact of imbalanced subsequent therapy on OS outcomes cannot be excluded. Therefore, the OS findings should be interpreted with caution, and differences in treatment patterns after progression between the two groups may partly explain why the OS interaction did not reach significance. Second, the small sample size of group C (20 patients per arm) limited statistical power, and the results for this subgroup should be regarded as exploratory; validation in larger cohorts is needed. And, group B comprised biologically heterogeneous non-driver alterations, with TP53 being the most frequent (36.8%). Due to limited sample sizes within individual genomic alterations, we were unable to perform alteration-specific analyses to determine whether the observed benefit in group B was driven by TP53 or other specific alterations, and the generalizability of the group B findings to individual non-driver alterations remains uncertain. Third, three different third-generation TKIs (osimertinib, aumolertinib, and furmonertinib) were pooled in this study, and chemotherapy exposure in the combination arm also varied with respect to platinum agent (carboplatin in 83.5% vs. cisplatin in 16.5%), number of cycles (median 4, IQR 4–6), and receipt of pemetrexed maintenance (69.5%). Although multivariable Cox regression showed no significant effect of TKI type on PFS or OS (all *P* > 0.05), and the post-PSM distribution of TKIs was balanced between groups, differences in pharmacokinetics, intracranial penetration, and chemotherapy intensity among these regimens may have influenced the results. Due to the limited sample size within each TKI subtype and chemotherapy stratum, formal sensitivity analyses by TKI type or chemotherapy exposure were not performed; such analyses should be undertaken in future studies with larger cohorts. Fourth, the definition and classification of co-alterations were based on the existing literature and clinical experience, and different NGS panels may vary in the number of genes tested and variant interpretation criteria, potentially affecting the reproducibility of the results. In addition, approximately 27% of patients underwent plasma rather than tissue NGS. Plasma ctDNA testing may have reduced sensitivity for detecting copy-number alterations and subclonal mutations compared with tissue NGS, which could lead to underestimation of co-alteration burden and potential misclassification of subgroup assignment. Due to the limited sample size, we were unable to perform a sensitivity analysis restricted to tissue NGS to confirm the robustness of the genomic classification; such an analysis should be undertaken in future studies with larger tissue-based cohorts. Fifth, this study was conducted in a Chinese population, and the applicability to Western populations, in whom the incidence of EGFR mutations is lower, requires further validation. Sixth, radiotherapy exposure was not systematically recorded in this retrospective cohort. Although radiotherapy was not part of the defined study intervention, some patients—particularly those with baseline brain metastases—may have received cranial radiotherapy, which could independently influence intracranial disease control and confound the interpretation of intracranial progression-free survival. In addition, symptomatic CNS disease at baseline and prior CNS-directed therapy (including neurosurgery) were not systematically documented. The potential impact of imbalanced prior CNS-directed therapy and symptomatic CNS disease between treatment groups on the intracranial efficacy findings therefore cannot be excluded. Future prospective studies with systematic documentation of radiotherapy exposure, prior CNS-directed therapy, and symptomatic CNS disease are warranted to clarify their independent contribution to intracranial efficacy outcomes. Seventh, we did not perform dynamic genomic profiling before treatment and after progression, which precludes direct elucidation of the specific molecular mechanisms by which combined chemotherapy delays resistance. Additionally, all primary analyses were based on propensity score matching, and alternative propensity-based sensitivity analyses such as inverse probability of treatment weighting (IPTW) were not performed. Although PSM achieved excellent covariate balance, future studies employing complementary propensity-based methods would further strengthen the robustness of the findings.

## 5 Conclusion

The present study demonstrates that baseline genomic co-alteration patterns are associated with the magnitude of benefit from first-line TKI plus chemotherapy in patients with advanced EGFR-mutant NSCLC. Patients harboring non-driver co-mutations may derive greater benefit from combination therapy, whereas those with EGFR mutation alone achieve favorable outcomes with TKI monotherapy. However, given the retrospective design, the biological heterogeneity within co-alteration subgroups, the exploratory nature of the small driver co-mutation subgroup, and the non-significant treatment-by-co-alteration interaction for OS, these findings should be regarded as hypothesis-generating. Prospective validation is required before co-alteration profiling can be integrated into pretreatment evaluation to guide individualized first-line treatment selection.

## Data Availability

The original contributions presented in the study are included in the article/Supplementary Material, further inquiries can be directed to the corresponding author/s.

## Glossary

AE,: adverse event
ALK,: anaplastic lymphoma kinase
ALT,: alanine aminotransferase
AMP,: association for molecular pathology
ARMS-PCR,: amplification refractory mutation system polymerase chain reaction
AST,: aspartate aminotransferase
AUC,: area under the curve
CAP,: college of American Pathologists
CI,: confidence interval
CIF,: cumulative incidence function
CNS,: central nervous system
CONSORT,: consolidated standards of reporting trials
CR,: complete response
CSCO,: Chinese society of clinical oncology
CT,: computed tomography
CTCAE,: common terminology criteria for adverse events
DCR,: disease control rate
ECOG,: Eastern cooperative oncology group
ECOG PS,: Eastern cooperative oncology group performance status
EGFR,: epidermal growth factor receptor
HR,: hazard ratio
IASLC,: international association for the study of lung cancer
iDCR,: intracranial disease control rate
iORR,: intracranial objective response rate
iPFS,: intracranial progression-free survival
IQR,: interquartile range
MRI,: magnetic resonance imaging
NGS,: next-generation sequencing
NSCLC,: non-small-cell lung cancer
ORR,: objective response rate; OS, overall survival
PD,: progressive disease
PFS,: progression-free survival
PR,: partial response
PSM,: propensity score matching
RECIST,: response evaluation criteria in solid tumors
SAE,: serious adverse event
SD,: stable disease
sHR,: subdistribution hazard ratio
SMD,: standardized mean difference
STROBE,: strengthening the reporting of observational studies in epidemiology
TKI,: tyrosine kinase inhibitor.
